# EGFR signaling and pharmacology in oncology revealed with innovative BRET-based biosensors

**DOI:** 10.1038/s42003-024-05965-5

**Published:** 2024-03-01

**Authors:** Florence Gross, Arturo Mancini, Billy Breton, Hiroyuki Kobayashi, Pedro Henrique Scarpelli Pereira, Christian Le Gouill, Michel Bouvier, Stephan Schann, Xavier Leroy, Laurent Sabbagh

**Affiliations:** 1 Domain Therapeutics North America Inc., 7171 Frederick-Banting, Saint-Laurent, Quebec H4S 1Z9 Canada; 2 Institute for Research in Immunology and Cancer, and Department of Biochemistry and Molecular Medicine, University of Montreal, 2950 Chemin de Polytechnique, Montreal, Quebec H3T 1J4 Canada; 3 Domain Therapeutics SA, 220 Boulevard Gonthier D’Andernach, 67400 Strasbourg-Illkirch, France

**Keywords:** Assay systems, Drug development, Translational research

## Abstract

Mutations of receptor tyrosine kinases (RTKs) are associated with the development of many cancers by modifying receptor signaling and contributing to drug resistance in clinical settings. We present enhanced bystander bioluminescence resonance energy transfer-based biosensors providing new insights into RTK biology and pharmacology critical for the development of more effective RTK-targeting drugs. Distinct SH2-specific effector biosensors allow for real-time and spatiotemporal monitoring of signal transduction pathways engaged upon RTK activation. Using EGFR as a model, we demonstrate the capacity of these biosensors to differentiate unique signaling signatures, with EGF and Epiregulin ligands displaying differences in efficacy, potency, and responses within different cellular compartments. We further demonstrate that EGFR single point mutations found in Glioblastoma or non-small cell lung cancer, impact the constitutive activity of EGFR and response to tyrosine kinase inhibitor. The BRET-based biosensors are compatible with microscopy, and more importantly characterize the next generation of therapeutics directed against RTKs.

## Abstract (web summary)

BRET-based biosensors allow the quantitative live-cell monitoring of RTKmediated signaling, revealing ligand-specific spatiotemporal and mutation-specific signaling biases, advancing the understanding of RTK functional selectivity.

## Introduction

Since their discovery, receptor tyrosine kinases (RTKs) have emerged as key regulators of cell growth, metabolism, differentiation, and survival. Consequently, dysregulated RTK activity often contributes to various disorders—most notably cancer—thus making this family of receptors prime therapeutic targets. RTK signaling and activity is classically described to follow the canonical model where ligand binding on the cell surface favors receptor dimerization and/or oligomerization, inducing a conformational change that culminates in the activation of the kinase domain. This event leads to *trans* autophosphorylation of receptor tyrosine residues, which serve as docking platforms for various signaling proteins containing Src homology-2 (SH2) and/or phospho-tyrosine-binding (PTB) domains. These domains bind to specific phospho-tyrosine residues within the receptor cytosolic tail and engage downstream mediators that propagate critical cellular signaling events^1^.

It is now evident that RTK signaling in physiological and pathological states is much more complex and multifaceted than initially described. Indeed, ever-increasing data is revealing that different ligands can stabilize distinct active conformational states of the same receptor, leading to different signaling and biological outcomes^2–5^. This concept, known as functional selectivity, has been well described for GPCRs and allows for selective modulation of receptor-downstream signaling networks^4,6–8^. Furthermore, we now know that many activated RTKs trigger signaling events from various endosomal compartments, which potentially contribute to drug resistance^9–11^.

Our understanding underlying the activity and pharmacology of RTKs remains incomplete, and their full therapeutic potential is largely underexploited. Such gaps are, in part, attributable to technological shortcomings in profiling the activation signature of the receptors. Indeed, many of the methodologies currently used to study RTK activation and screen for therapeutics targeting RTKs revolve around the kinase activity. Kinase activity-based assays are limited in throughput and can overlook additional key determinants of ligand therapeutic efficacy, including kinetics of responses, cellular localization, and functional selectivity.

We present herein live-cell bioluminescence resonance energy transfer (BRET)-based biosensors allowing for real-time spatiotemporal monitoring of RTK signaling across more than 10 effector proteins/pathways. The RTK bioSens-All® platform is built using enhanced bystander BRET, or ebBRET^12^, which exploits the ability of luciferase and GFP from *Renilla reniformis* to self-associate with moderate affinity and optimally transfer energy^13^. Using these naturally interacting chromophores, we developed biosensors that allow for quantitative live-cell monitoring of RTK-mediated and -specific trafficking of SH2 domain-containing protein to the plasma membrane and early endosomes following receptor activation. We used EGFR as a model receptor to demonstrate the spectrum of applications of our biosensors in studying RTK biology and pharmacology. The results obtained replicated the expected receptor coupling profiles and pharmacology while revealing novel ligand-specific spatiotemporal signaling biases and mutation-specific signaling signatures. The selectivity and sensitivity of the biosensors provide unmatched tools for both spectrometric and microscopy-based spatiotemporal studies of RTK signaling, which is also compatible with high-throughput screening for novel therapeutics.

## Results

### Characterization of RTK signaling at the plasma membrane using effector-specific ebBRET-based biosensors

**Fig. 1:**
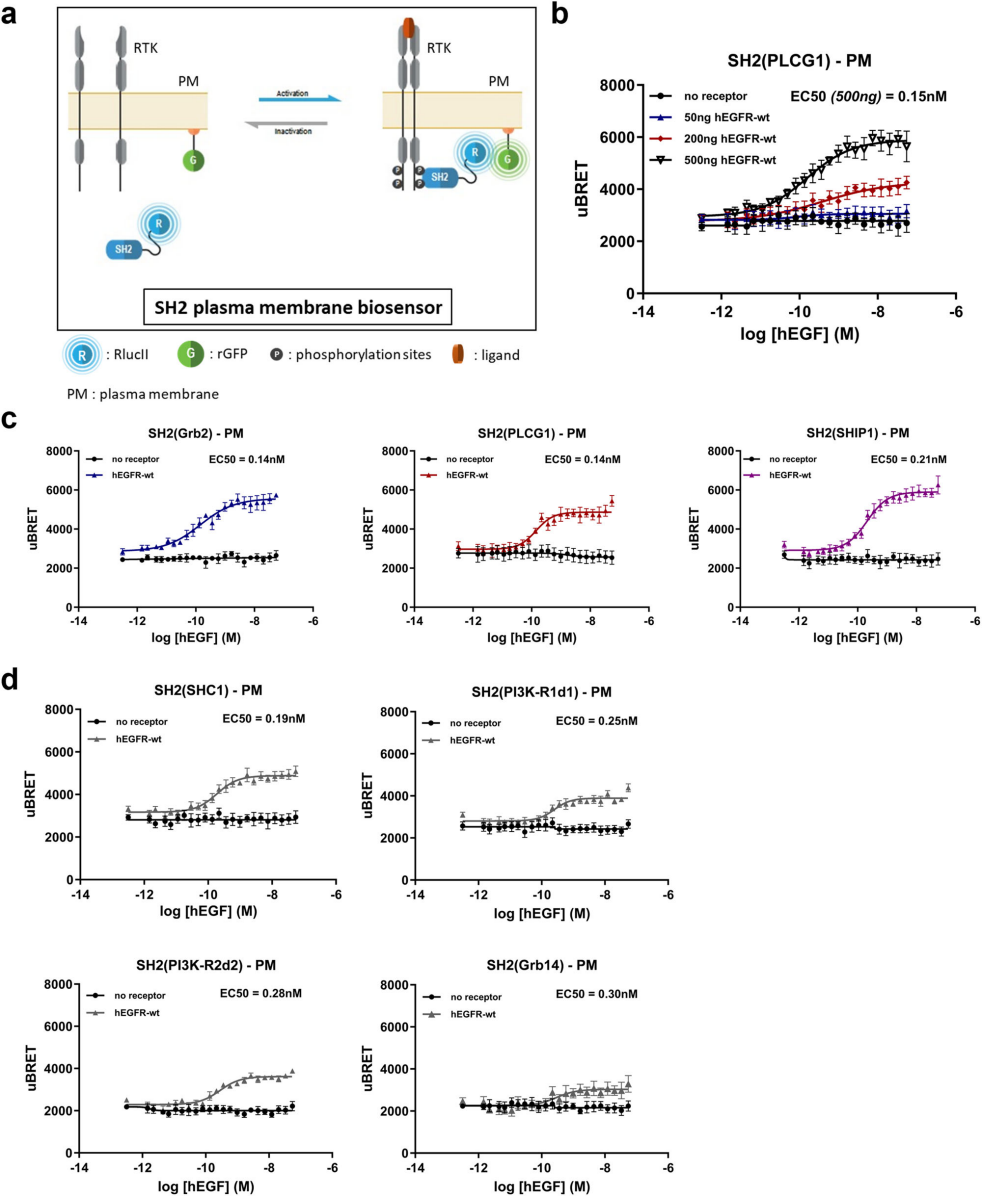
Overview and pharmacological validation of RTK biosensors at the plasma membrane (PM). **a** Schematic representation of the ebBRET-based assays to monitor the recruitment of RlucII-SH2 effectors to the PM. Upon ligand binding to the RTK, RlucII-fused SH2-domain effectors are recruited to phospho-tyrosine sites on the RTK C-terminal tail. This allows an energy transfer between the RlucII-SH2 effector and the PM-anchored rGFP leading to an increase in the BRET signal. **b** HEK293 cells transfected with different amounts of EGFR-WT-coding plasmid were exposed to increasing concentrations of EGF for 10 min to monitor ligand-induced RlucII-SH2(PLCG1) recruitment to the PM. **c** EGF concentration response curves for RlucII-SH2 effector recruitment to the PM in HEK293 cells co-transfected with various SH2(Grb2), SH2(PLCG1), and SH2(SHIP1) RlucII-SH2 effectors or **d** SH2(SHC1), SH2(PI3K-R1d1), SH2(PI3K-R2d2), and SH2(Grb14) RlucII-SH2 effectors with EGFR-WT or no receptor control. Results were obtained after a 10-minute stimulation with EGF and are expressed as uBRET (mean ± SEM; *n* = 3–4 independent experiments). BRET values were obtained by calculating the ratio of the light emitted by the energy acceptor (rGFP; 515 ± 20 nm)) over the light emitted by the energy donor (RlucII; 400 nm ± 70 nm). BRET ratios were then normalized to uBRET = ((BRET ratio – A)/(B − A)) * 10,000. Constants A and B correspond to the following values: A = pre-established BRET ratio obtained from transfection of negative control (vector coding for RlucII alone). B = pre-established BRET ratio obtained from transfection of positive control (vector coding for a GFP10-RlucII fusion protein).

Phosphorylation of tyrosine residues in the cytosolic domain of RTKs leads to the recruitment of SH2 domain-containing proteins. SH2 domains represent the largest class of phospho-tyrosine-selective recognition domains in the human proteome^14^, with over one hundred SH2-domain-containing proteins described in humans^15^. We used the SH2 domains of seven RTK-interacting effectors to generate seven *Renilla Reniformis* luciferase (RlucII)-fused SH2 as the energy donor component of the biosensors (Supplementary Fig. 1). The SH2 domains selected have been reported to interact with a range of RTKs in a pTyr motif-specific manner^16^. We used *Renilla Reniformis* GFP (rGFP)-fused to the CAAX motif of K-Ras as the BRET acceptor, thus targeting rGFP to the plasma membrane (PM)^12^ (Fig. 1a). The rGFP-CAAX fusion thereby allowed us to assess RTK effector engagement at the PM and more importantly, permitted to work with unmodified (i.e., untagged) receptors.

To evaluate if responses were receptor concentration-dependent, we performed receptor titration studies using EGFR as a model system. We monitored ligand-induced RlucII-SH2(PLCG1) recruitment to the PM following a 10-min stimulation with human EGF (hEGF) in HEK293 transiently transfected with different amounts of human EGFR coding plasmid (Fig. 1b). Receptor concentration-dependent and ligand concentration-dependent increase in BRET signals were observed following ligand addition. No effect was observed in the absence of EGFR transfection, confirming the specificity of the response. The largest assay window was obtained with 500 ng of EGFR; this quantity of receptor was therefore used for all subsequent experiments.

To further investigate EGFR signaling, we compared the responses of biosensors based on three different SH2-domain-containing biosensors in HEK293 cells expressing or not EGFR. The biosensors tested are generally (but not exclusively) associated with the activation of three major RTK signaling axes: SH2(Grb2) for the MAPK pathway, SH2(PLCG1) for the PKC pathway, and SH2(SHIP1) for the Akt pathway (non-canonical activation mechanism). The measured response demonstrated an EGFR and hEGF ligand concentration-dependent BRET signals with EC_50s_ of 0.14 nM, 0.14 nM, and 0.21 nM, respectively (Fig. 1c). We also validated the specificity of four other RlucII-tagged SH2 domain biosensors from the following RTK-interacting proteins: SHC1, PI3K-R1d1, PI3K-R2d2 and Grb14. Ligand concentration-dependent responses were only observed in cells expressing EGFR and the presence of hEGF (EC_50_ between 0.1 and 0.3 nM) (Fig. 1d). Interestingly, Hill coefficients differed for each SH2 domain biosensor: 0.74 (SH2(Grb2)), 1.48 (SH2(PLCG1)), 1.23 (SH2(SHIP1)), 1.31 (SH2(SHC1)), 1.67 (SH2(PI3K-R1d1)), 1.34 (SH2(PI3K-R2d2)), and 1.35 (SH2(Grb14)). This may be suggestive of differential binding cooperativity between each molecule of RlucII-tagged SH2 domain protein and/or between the latter and endogenous effectors that interact with various pTyr motifs in the hEGFR cytoplasmic domain.

### Spectroscopic and microscopic assessment of ligand-specific EGFR signaling at the plasma membrane

**Fig. 2:**
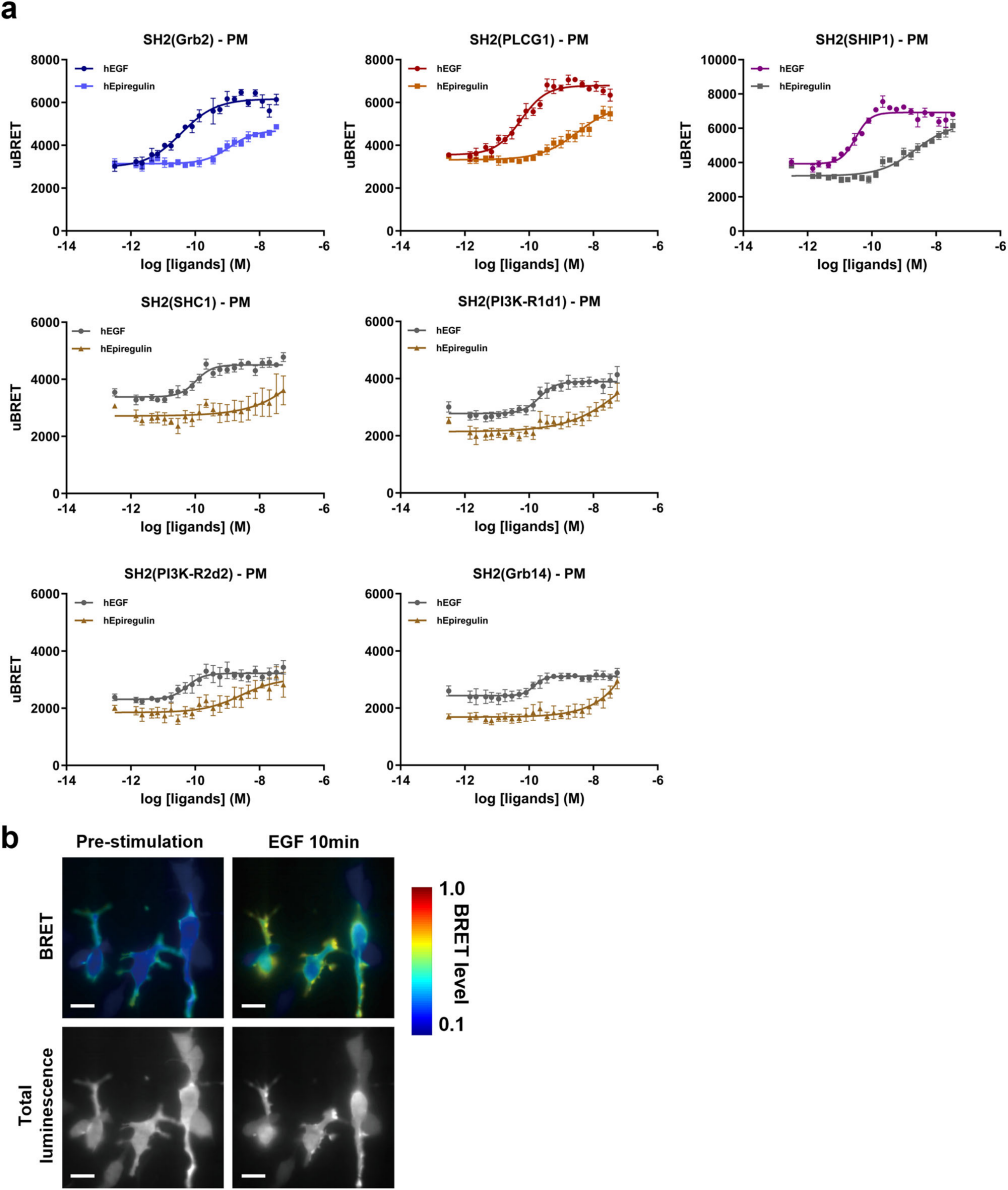
Profiling of EGFR ligands—recruitment of SH2 biosensors to the plasma membrane (PM). HEK293 cells were co-transfected with plasmids coding for EGFR-WT, rGFP-CAAX, and the indicated RlucII-tagged, effector-specific SH2 domains. **a** EGF and Epiregulin concentration response curves for RlucII-SH2 effector recruitment to the PM in HEK293 cells co-transfected with various RlucII-SH2 effectors and EGFR-WT. Results were obtained after a 10-minute stimulation with EGF or Epiregulin and are expressed as uBRET (mean ± SEM; *n* = 3-4 independent experiments). Details about the calculation of uBRET are provided in the “Calculations and statistical analysis” of the Materials and methods section. **b** Imaging ebBRET to monitor membrane translocation of RlucII-SH2(Grb2). BRET and total luminescence images were obtained in the presence of 10 µM Coelenterazine-400a. The first and second columns are images from the same field of view before and after a 10-minute stimulation with 10 nM EGF, respectively. In each image, BRET levels correspond to the ratio of acceptor (rGFP) photon counts to donor photon counts calculated for each pixel^63,66^. BRET levels are expressed as a color-coded heat map, with the lowest (0.1) being black and purple and the highest (1.0) being red and white. Scale bars, 20 µm.

Using the panel of seven biosensors validated above, we then compared the ability of two natural EGFR ligands, hEGF and hEpiregulin, to activate the receptor by monitoring BRET signals. Plasma membrane recruitment of these biosensors was recorded following stimulation with increasing concentrations of each ligand. hEGF promoted the recruitment of the seven biosensors to the PM with similar potencies (EC_50_ ranging from 3 × 10^−11^ M to 10 × 10^−11^ M) (Fig. 2a). Compared to hEGF, hEpiregulin was at least 100-fold less potent in promoting the recruitment of SH2 effectors to EGFR’s tyrosine-phosphorylated sites based on BRET measurements (Fig. 2a). The sensitivity of the SH2-based biosensors allowed the quantification of responses to different ligands.

Recent advances in microscopy and more efficient BRET probes, such as those offered by ebBRET brought new opportunities for the development of BRET-based imaging tools. BRET imaging offers the possibility to validate the localization of newly designed ebBRET biosensors and to visually assess BRET signals in subcellular compartments. HEK293 cells were co-transfected with the EGFR receptor and the RlucII-tagged effector, SH2(Grb2), to monitor its recruitment at the rGFP-tagged PM. BRET images were first obtained in resting conditions and 10 min after stimulation with 10 nM hEGF. As shown in Fig. 2b and Supplementary Movie 1, the recruitment of the SH2(Grb2) effector to the PM upon hEGF stimulation using ebBRET-based imaging was sustained for at least 1 h. These BRET imaging results strongly support the data generated using spectrometric methods (Fig. 2a) while also illustrating the possibility of transposing the biosensor platform to microscopy (Fig. 2b).

### Spectroscopic and microscopic assessment of ligand-specific EGFR signaling at the early endosomes

**Fig. 3:**
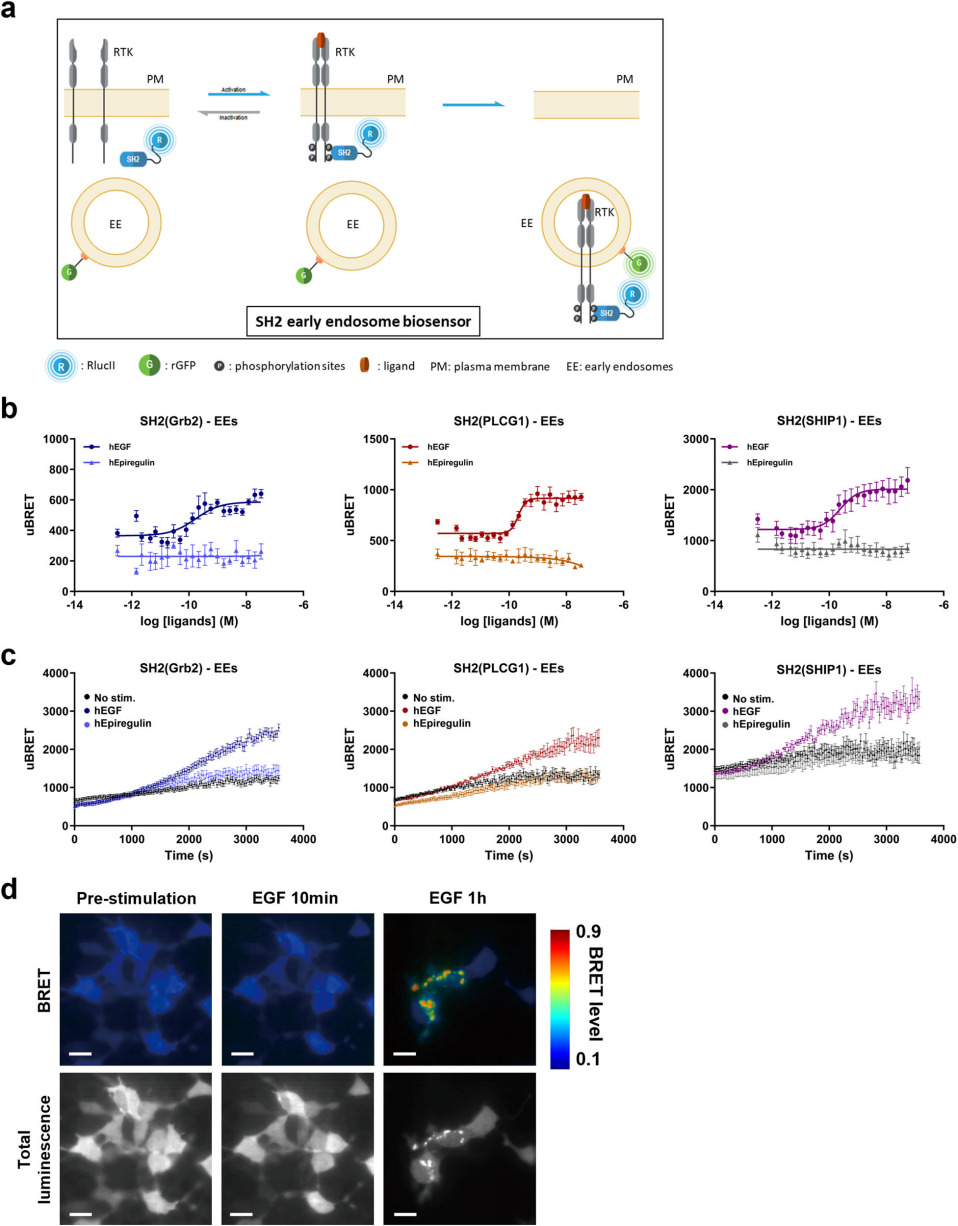
Ligand-biased trafficking of SH2 biosensors to early endosomes (EEs). **a** Schematic representation of the ebBRET-based assays to monitor the trafficking of RlucII-SH2 effectors to EEs. Translocation of RlucII-SH2 effectors is followed into rGFP-tagged EEs, leading to an increase in BRET signal, allowed by an energy transfer between the RlucII-SH2 effector and the EEs-anchored rGFP. **b** HEK293 cells were co-transfected with plasmids coding for EGFR-WT, rGFP-FYVE, and the indicated RlucII-tagged effector-specific SH2 domains. EGF and Epiregulin dose-dependent translocation of RlucII-SH2(Grb2), RlucII-SH2(PLCG1) and RlucII-SH2(SHIP1) to EEs after a 60-minute stimulation. **c** Real-time trafficking of the same three biosensors as in (**b**) to EEs. Cells were stimulated with an EC_80_ of EGF or a maximal concentration of Epiregulin, and the BRET signal was immediately recorded every 30 s for a period of 60 min. All results are expressed as uBRET (mean ± SEM; *n* = 3–4 independent experiments). Details about the calculation of uBRET are provided in the “Calculations and statistical analysis” of Materials and methods section. **d** Imaging ebBRET to monitor the translocation of RlucII-SH2(Grb2) into early endosomes. BRET and total luminescence images were obtained in the presence of 10 µM Coelenterazine-400a. The first and second columns are images from the same field of view before and after a 10-min stimulation with 10 nM EGF, respectively. The third column is from a different field of view, imaged after a 60-min incubation with 10 nM EGF. In each image, BRET levels correspond to the ratio of acceptor (rGFP) photon counts to donor photon counts calculated for each pixel^63,66^. BRET levels are expressed as a color-coded heat map, with the lowest (0.1) being black and purple and the highest (0.9) red and white. Scale bars, 20 µm.

To assess whether EGFR activation by hEGF and hEpiregulin could be detected in early endosomes (EEs) we used rGFP-fused to the FYVE domain of endofin as BRET acceptor. Specific co-localization of the rGFP-FYVE fusion construct used in the present study to EEs (Rab5+ compartment) has been previously confirmed by microscopy^12^ using identical conditions. Targeting rGFP to EE allowed us to detect RTK effector engagement at the EE compartment by measuring BRET with the RlucII-tagged effectors (Fig. 3a). SH2(Grb2), SH2(PLCG1), and SH2(SHIP1) translocation to EEs was examined after a 60-minute stimulation with increasing concentrations of hEGF or hEpiregulin. For hEGF, all SH2 biosensors studied translocated to EEs with EC_50_s between 1.7 × 10^−10^ M and 2.4 × 10^−10^ M. In contrast, no responses were obtained upon hEpiregulin stimulation (Fig. 3b). These results are consistent with published evidence showing that hEpiregulin does not promote EGFR internalization to EEs^17,18^.

To validate that we were in the appropriate time window for translocation to the EEs after stimulation with hEpiregulin, we examined in real-time the trafficking of the three biosensors to EEs. Cells were stimulated with hEGF or hEpiregulin and BRET signals were monitored for a period of 60 min; measurements were recorded every 30 s. We observed again that hEGF, but not hEpiregulin, promoted a time-dependant increase of SH2(Grb2), SH2(PLCG1), and SH2(SHIP1) biosensors localization to the EEs, with the appearance in the EEs after 20 min of ligand stimulation (Fig. 3c). Altogether, these results highlight the capacity of the biosensors to monitor the spatiotemporal ligand signaling bias following EGFR activation.

BRET imaging was used to confirm the above observations for hEGF-induced SH2(Grb2) translocation to EEs (Fig. 3d). BRET signals were imaged before and at 10 and 60 min after hEGF treatment (10 nM). No signal was detected following a 10 min stimulation but a robust increase in BRET signal was observed in punctate intracellular structures after 60 min of stimulation in agreement with the spectrometric kinetic measurements.

Altogether, the newly generated RlucII-SH2 biosensors allowed the assessment of signaling kinetics in various cellular compartments and timescales ranging from milliseconds to hours and using BRET microscopy.

### Spatiotemporal analysis of EGFR signaling blockade with the EGFR tyrosine kinase inhibitor Gefitinib

**Fig. 4:**
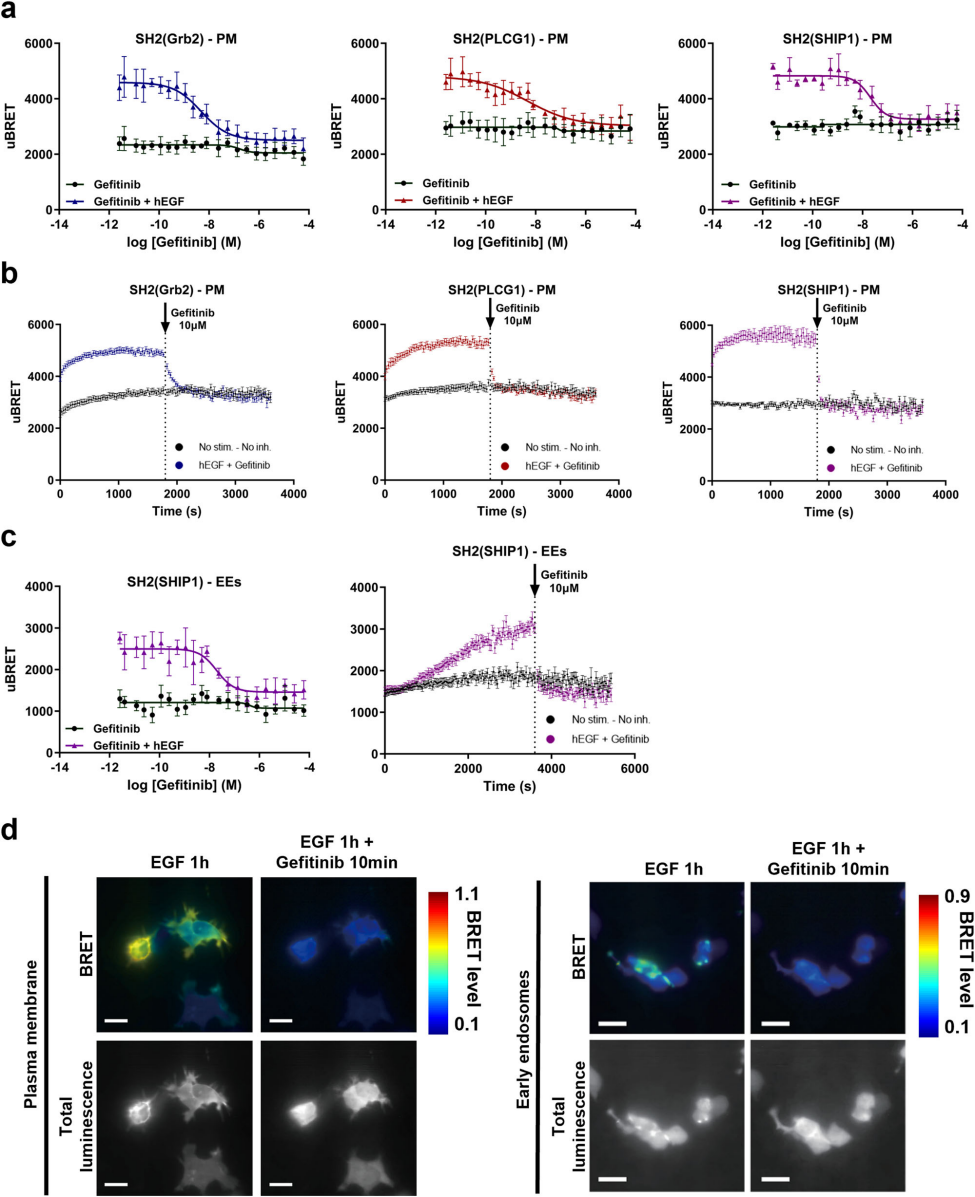
Inhibitory effects of Gefitinib on EGFR-mediated signaling at the plasma membrane (PM) and early endosomes (EEs). HEK293 cells were co-transfected with plasmids encoding EGFR-WT, the indicated RlucII-tagged effector-specific SH2 domains, and either rGFP-CAAX (**a**, **b**, and **d**) or rGFP-FYVE (**c**, **d**). **a** Cells were pretreated during 30 min with increasing concentrations of Gefitinib and BRET signal was measured to detect potential agonist activity. Cells were then stimulated for 10 min with an EC_80_ of EGF, and the BRET signal was measured to detect the inhibitory effects of Gefitinib. **b** For the real-time kinetics analysis of Gefitinib’s inhibitory activity on EGFR-downstream effector recruitment to the PM, transfected cells were stimulated with an EC_80_ of EGF, and BRET signal was immediately measured every 30 s for a period of 30 min. EGF-stimulated cells were then treated with 10 µM of Gefitinib, and the BRET signal was recorded for an additional 30 min to reveal Gefitinib’s inhibitory activity. **c** To determine the impact of Gefitinib on EGFR-WT promoted translocation of RlucII-SH2(SHIP1) to the EEs, cells were treated as in (**a**) and (**b**) (for static and real-time kinetics measurements, respectively) with the exception that stimulation with EGF was conducted for 60 min to ensure sufficient RlucII-SH2(SHIP1) translocation to the EEs. All data are expressed as uBRET (mean ± SEM; *n* = 3–4 independent experiments). Details about the calculation of uBRET are provided in the “Calculations and statistical analysis” of Materials and Methods section. **d** Imaging ebBRET to monitor the inhibitory effects of Gefitinib in membrane translocation or into early endosomes of RlucII-SH2(Grb2). BRET and total luminescence images were obtained in the presence of 10 µM Coelenterazine-400a. The first and second columns show pretreated cells with 10 nM EGF for 1 h, imaged before and after a 10-minute incubation with 1 µM Gefitinib, either at the PM or at the EEs. In each image, BRET levels correspond to the ratio of acceptor (rGFP) photon counts to donor photon counts calculated for each pixel^63,66^. BRET levels are expressed as a color-coded heat map, with the lowest (0.1) being black and purple and highest (1.1) red and white. Scale bars, 20 µm.

To further demonstrate the specificity of the SH2-based biosensor BRET signals, we monitored the inhibitory action of Gefitinib (Iressa^TM^), an EGFR-specific tyrosine kinase inhibitor (TKI), commonly used as a first-line treatment for some breast and lung cancers. Cells expressing three separate SH2-biosensors were treated with increasing concentrations of Gefitinib for 30 min, followed by hEGF for 10 min. Gefitinib inhibited the recruitment of SH2(Grb2), SH2(PLCG1), and SH2(SHIP1) to the PM, with observed IC_50_s of 4 nM, 5 nM, and 21 nM respectively (Fig. 4a). Of note, these values are comparable to previously reported data in the literature using other methods (reported IC_50_s comprised between 30 nM and 40 nM)^19,20^. We also evaluated Gefitinib’s inhibitory activity in real time. Responses were rapidly reversed within 180 s, 90 s, and 60 s for SH2(Grb2), SH2(PLCG1), and SH2(SHIP1), respectively, upon the addition of Gefitinib (10 μM) after 30 min of stimulation with hEGF (Fig. 4b).

An understanding of trafficking and signaling of RTKs in the EEs is important since it has been proposed to be an important mechanism contributing to drug resistance^21–23^. It is thus necessary to properly examine and define the effects of therapeutics in such intracellular compartments. For this purpose, we measured BRET in the EEs using the SH2(SHIP1) biosensor, as this biosensor displayed the greatest assay window in that compartment. After a 30-minute incubation with increasing concentrations of Gefitinib, cells were stimulated for 60 min with hEGF (55 nM) to promote effector recruitment to EEs. As shown in Fig. 4c, Gefitinib was able to reverse the recruitment of SH2(SHIP1) to the EEs with a similar potency to the one observed at the PM (IC_50_ = 20 nM). To examine the real-time recruitment of SH2(SHIP1) to the EEs, EGFR-expressing cells were stimulated for 60 min with hEGF before the addition of a 10 µM Gefitinib (Fig. 4c). As observed at the PM, the signal was rapidly reversed within less than 60 s, showing the capacity of this TKI to inhibit the recruitment of SH2 effectors at both the PM and EEs.

BRET imaging was used to confirm the inhibitory effects of Gefitinib on hEGF-induced SH2(Grb2) recruitment at the PM and EEs (Fig. 4d and Supplementary Movie 1). BRET signals were assessed following the addition of 1 µM of Gefitinib after 1 h of stimulation with 10 nM hEGF, either looking at the PM or at EEs. BRET signals were completely reversed in both compartments with Gefitinib, further highlighting the advantage of using SH2-biosensors to characterize the activity of TKIs in different cellular compartments.

### Endogenous EGFR signaling in cancer cell lines

**Fig. 5:**
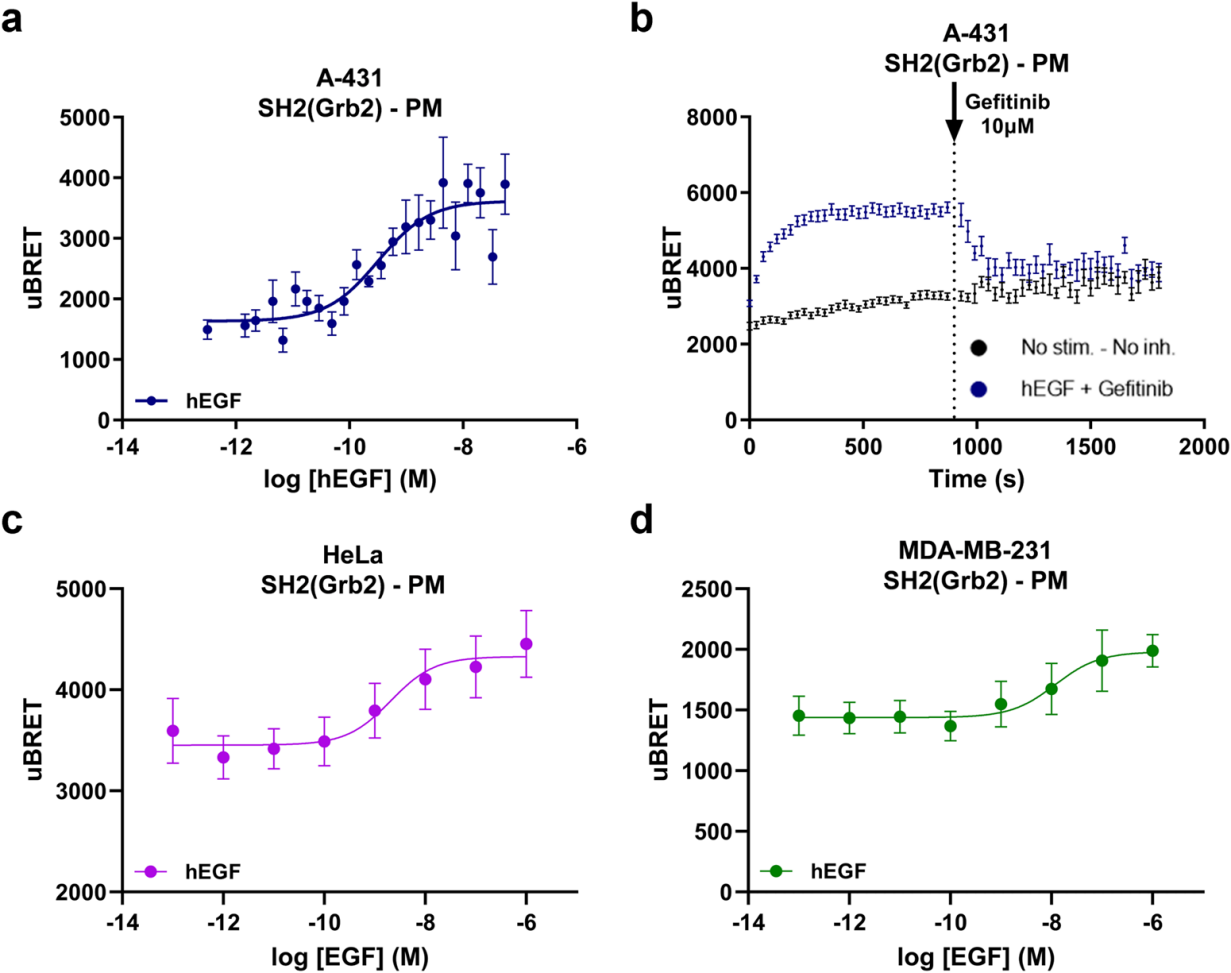
Measurement of endogenous EGFR signaling at the plasma membrane (PM) in A-431, HeLa, and MDA-MB-231 cells. A-431, HeLa, and MDA-MB-231 cells were transiently co-transfected with rGFP-CAAX- and RlucII-SH2(Grb2)-encoding plasmids. **a** EGF dose-dependent engagement of RlucII-SH2(Grb2) following 10 min of stimulation in A-431 cells. **b** Real-time kinetics of RlucII-SH2(Grb2) PM recruitment after stimulation of A-431 cells with an EC_80_ of EGF. BRET was recorded every 30 s following the addition of EGF. After 15 min of stimulation with EGF, cells were treated with 10 µM of Gefitinib for an additional 15 min. **c**, **d** EGF dose-dependent engagement of RlucII-SH2(Grb2) following 30 and 2 min of stimulation in (**c**) HeLa and (**d**) MDA-MB-231 cell, respectively. Data are expressed as uBRET (mean ± SEM; *n* = 3–4 independent experiments). Details about the calculation of uBRET are provided in the “Calculations and statistical analysis” of the Materials and methods section.

A-431 human epidermoid carcinoma cells are known to express high levels of EGFR and are often used to study cancer-associated cell signaling events. We thus used this cell line to examine the ability of ebBRET-based RTK biosensors to measure signaling downstream of endogenously expressed RTKs. A-431 cells were transfected with the SH2(Grb2) effector and its recruitment to the PM was measured following stimulation of endogenous EGFR with hEGF (Fig. 5a). Stimulation of transfected A-431 cells produced a SH2(Grb2) response similar to what was observed in EGFR-transfected HEK293 cells (i.e., potency: EC_50_ = 0,32 nM vs 0,14 nM; efficacy: *E*_max_ = 4000 vs. 5700). We then evaluated the recruitment of SH2(Grb2) to the PM in real-time after addition of hEGF and its subsequent inhibition following the addition of 10 µM Gefitinib after 15 min of hEGF stimulation. SH2(Grb2) was recruited to the PM within 4 min and its activation was reversed by Gefitinib within 3 min (Fig. 5b). In addition to A-431 cells, which express high levels of EGFR, we tested the capacity of our SH2 biosensors to detect endogenous EGFR signaling in cells expressing lower receptor levels of the receptor (i.e., in HeLa and MDA-MB-231 cells). As shown in Fig. 5c, d, EGF dose-dependently led to EGFR activation and PM recruitment of the SH2(Grb2) biosensor in both cell lines. These data highlighted the capability of ebBRET-based RTK sensors to be used in different pathophysiologically relevant cellular models and demonstrate the sensitivity of the assays to detect signaling events from endogenously expressed RTKs.

### Impact of cancer-associated EGFR point and truncation mutations on EGFR response

**Fig. 6:**
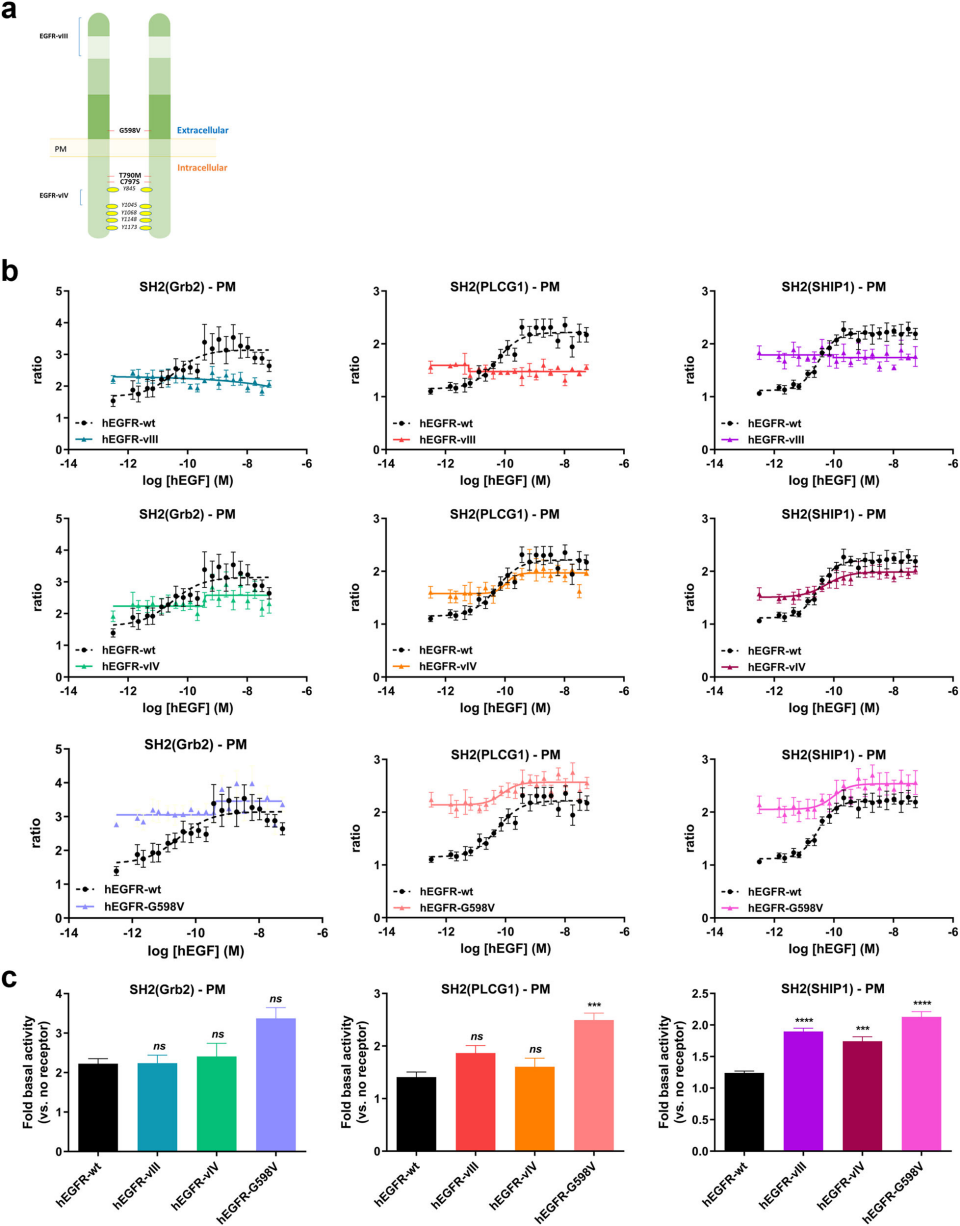
Study of the effects of various EGFR mutations on pathway-specific ligand-induced signaling and constitutive receptor activity at the plasma membrane (PM). **a** Schematic representation of EGFR mutations frequently found in glioblastoma (GBM; *EGFR-vIII, -vIV*, and *-G598V)* and non-small-cell lung carcinoma (NSCLC; *EGFR-T790M, -C797S, T790M/C797S*). **b** HEK293 cells were co-transfected with plasmids encoding the indicated RlucII-SH2 effectors, rGFP-CAAX without or with a plasmid encoding either EGFR-WT, EGFR-vIII, -vIV, or -G598V mutants. Cells were stimulated with increasing concentrations of EGF for 10 min, and BRET was recorded thereafter. uBRET data obtained with EGFR-WT and EGFR-mutants were all normalized to the mean uBRET calculated for control wells present on the same assay plate (i.e., wells containing non-stimulated cells transfected solely with the indicated biosensor; EGFR was not transfected). Data are expressed as ‘Fold response vs. no receptor transfected cells (=1)’ calculated according to the following formula: uBRET experimental well/AVE uBRET plate-specific control wells. Data shown are the mean ± SEM for 3–4 independent experiments. EGFR-WT signaling profiles (in black) are reproduced on each mutant’s dose response curve for visual comparison. **c** To compare basal (constitutive) activities between EGFR-WT and various EGFR mutants at the plasma membrane, we used normalized uBRET data for the non-stimulated conditions shown in panel (**b**) (normalization method described in (**b**). Histograms were generated for the comparison of constitutive activities (mean ± SEM; *n* = 4 to 6; one-way ANOVA, Bonferroni post hoc adjustment for multiple comparisons: ***p < 0.001 and ****p < 0.0001 compared to respective WT basal). Data are expressed as ‘Fold basal activity (vs. no receptor)’.

Glioblastoma (GBM), a very aggressive form of brain cancer with poor prognosis^24^, presents multiple aberrant genomic alterations of EGFR that are responsible for disrupted signaling and resistance to therapies^25^. EGFR extracellular domain truncation mutations (i.e., EGFR-vI, -vII, and -vIII)^26,27^, deletion in the kinase domain (i.e., EGFR-vIV and –vV mutants)^28^ and extracellular point mutations (i.e., A298V, R324L and G598V)^29,30^ are exclusively found in GBM^31^ (Fig. 6a). Furthermore, it has been reported that mutations in EGFR can lead to significant changes in receptor constitutive activity^32–34^.

We sought to determine signaling profiles at the PM of three types of mutants recurrent in GBM: EGFR-vIII, -vIV, and –G598V. HEK293 cells were transfected with plasmids expressing each of the mutated forms of EGFR, and either RlucII-tagged effector SH2(Grb2), SH2(PLCG1), or SH2(SHIP1). The translocation of the effectors to the PM was then monitored upon stimulation with increasing concentration of hEGF (Fig. 6b). Our data revealed that EGFR-vIII did not respond to stimulation with hEGF compared to wild-type (WT) EGFR. These results validated structural data showing that the EGF binding site is located within the portion of the receptor deleted in the EGFR-vIII mutant^35^. However, the basal BRET signal was found to be increased compared to the WT receptor reflecting the constitutive activity of the receptor. When analyzing ligand-independent receptor activation due to the presence of mutations, our data demonstrated that EGFR–vIII displayed significant constitutive activation for SH2(SHIP1) biosensor but not SH2(Grb2) and SH2(PLCG1) biosensors (Fig. 6c). EGFR-vIV responded to hEGF stimulation but with a ~10-fold lower potency than that observed with WT EGFR with SH2(PLCG1) and SH2(SHIP1) biosensors (Fig. 6b). Loss of some tyrosine-phosphorylated sites^28^ may account for the above observations. Finally, EGFR-G598V, which is found in smaller cohorts of GBM^36^, showed similar maximal ligand responses compared to WT EGFR (Fig. 6b). Conversely, EGFR–vIV displayed significant ligand-independent constitutive activity with the SH2(SHIP1) biosensor only, while the EGFR-G598V point mutant displayed significant constitutive activity with SH2(PLCG1) and SH2(SHIP1) biosensors (Fig. 6c).

**Fig. 7:**
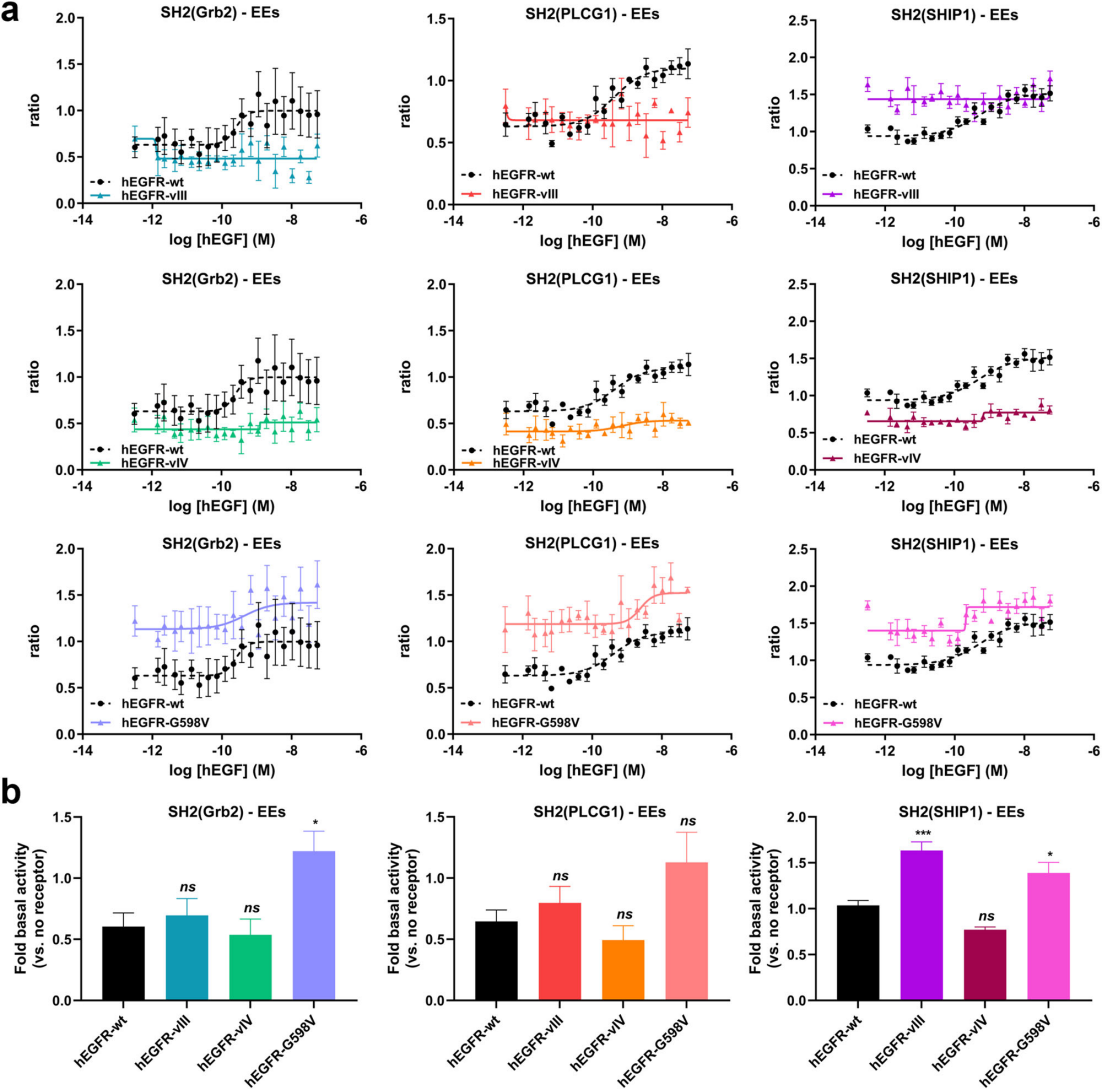
Study of the effects of various EGFR mutations on pathway-specific ligand-induced signaling and constitutive receptor activity at the early endosomes (EEs). **a** HEK293 were co-transfected with plasmids encoding the indicated RlucII-SH2 effectors, rGFP-FYVE without or with EGFR-WT, EGFR-vIII, -vIV, or -G598V mutants. Cells were stimulated with increasing concentrations of EGF for 60 min, and BRET was recorded thereafter. Data were normalized and represented as described in Fig. 6b and are the mean ± SEM for 3–4 experiments. EGFR-WT signaling profiles in the EEs (in black) are reproduced on each mutant’s dose response curve for visual comparison of differences in effectors’ translocation to the EEs. **b** To compare basal (constitutive) activities between EGFR-WT and various EGFR mutants at early endosomes, we used normalized uBRET data (normalization method described in Fig. 6b) from non-stimulated conditions shown in panel a. Histograms were generated for the comparison of constitutive activities (mean ± SEM; *n* = 3 to 4; one-way ANOVA, Bonferroni post hoc adjustment for multiple comparisons: *p < 0.05 and ***p < 0.001 compared to respective WT basal). Data are expressed as ‘Fold basal activity (vs. no receptor)’.

We then investigated the impact of EGFR-vIII, -vIV, and –G598V mutations on ligand-dependent and -independent activity in the EEs. Similar to its activity at the PM, EGFR-vIII induced selective constitutive SH2(SHIP1) effector recruitment to EEs; stimulation with EGF did not further enhance the translocation of effectors to EE (Fig. 7a, b). Interestingly EGFR-vIV displayed a complete lack of effector recruitment to EEs, despite being almost fully functional in recruiting the SH2 effectors to the PM (Fig. 7a). These observations may be linked to the fact that this mutant lacks the Y1045 phosphorylation site shown to promote receptor internalization and degradation through the recruitment of ubiquitin ligase c-CBL^37,38^. Alternatively, failure of the Y1045 mutant to recruit c-Cbl may indirectly impact EGFR trafficking by impairing ubiquitination (and subsequent activation) of (an) accessory protein(s) involved in receptor internalization as previously described^39^. Finally, the EGFR-G598V mutant displayed increased constitutive activity for both SH2(Grb2) and SH2(SHIP1) effectors at the EEs and still responded to hEGF stimulation in this compartment indicating that, similarly to the WT, EGFR-G598V is internalized upon agonist stimulation (Fig. 7a, b). Differences in EE SH2 effector recruitment to the various EGFR mutants is not explained by differences in receptor expression, as quantification of surface and total EGFR expression by flow cytometry demonstrated comparable or even slightly higher expression levels of EGFR mutants to EGFR-WT when overexpressed in HEK293 cells (Supplementary Figs. 2 and 3).

**Fig. 8:**
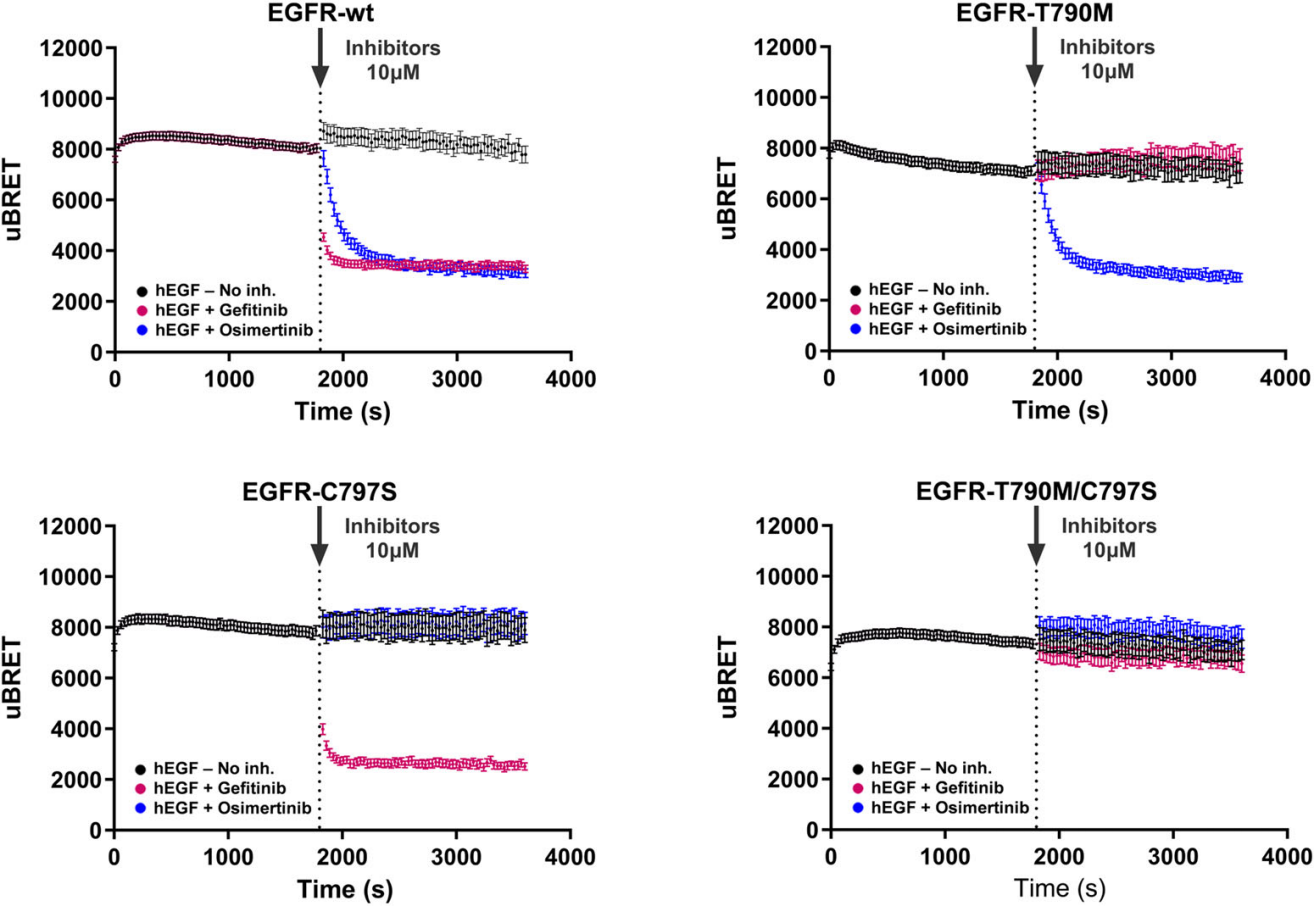
Real-time kinetic analysis of the inhibitory effects of tyrosine kinase inhibitors on EGFR mutants. HEK293 cells were co-transfected with plasmids encoding EGFR-WT or EGFR-T790M, -C797S, T790M/C797S mutants, along with rGFP-CAAX and RlucII-SH2(PLCG1). Cells were first stimulated with an EC_80_ of EGF, and BRET signals were immediately recorded every 30 s over a period of 30 min. Thereafter, 10 µM of the indicated tyrosine kinase inhibitor (Gefitinib or Osimertinib) was added to the wells, and BRET was immediately measured for an additional 30 min. All data are expressed as uBRET (mean ± SEM; *n* = 3–4 independent experiments).

EGFR point mutations EGFR-T790M, -C797S, and -T790M/C797S are acquired mutations that arise during treatment with first and third-generation EGFR TKIs and occur in non-small cell lung carcinoma (NSCLC)^40,41^. We assessed the inhibitory effects of two TKIs approved for the treatment of NSCLC on these EGFR mutants (Fig. 8). Looking at the real-time recruitment of SH2(PLCG1) effector to the PM, we followed the activation of the mutated receptors, compared to WT EGFR, after stimulation with hEGF. We then followed the reversal of effector engagement upon the addition of 10 µM of first-generation TKI, Gefitinib, or third-generation TKI, Osimertinib (Tagrisso^TM^). Interestingly, we observed different kinetics of inhibition between the two TKIs on WT EGFR. Gefitinib displayed faster inhibitory activity (within 60 s) compared to Osimertinib (5 min) in completely reversing WT EGFR signaling. Gefitinib reversed the activity of WT EGFR and –C797S, but this TKI was ineffective on the –T790M mutation.

These observations are supported by Gefitinib’s mode of action, which involves binding to the EGFR kinase ATP binding site and the fact that the –T790M mutation is located in the ATP binding site^42,43^. In contrast, Osimertinib reversed the activity of both EGFR-WT and –T790M, but not of –C797S mutant (Fig. 8). Osimertinib was developed to circumvent –T790M mutation as it forms a covalent bond with Cys797 at the edge of the ATP-binding pocket^44^. Its efficacy is, however, compromised by the presence of a mutation on this cysteine residue^45^. As expected, both TKIs were ineffective on the EGFR–T790M/C797S double mutant. Our observations are consistent with data in the literature, showing the capacity of the RTK biosensor platform to discriminate the effects of different TKIs, and the impact of mutations on TKI activity.

## Discussion

We have developed a BRET-based biosensor platform enabling the study of RTK activity in living cells. This platform of genetically encoded biosensors is based on ebBRET^12^, which confers the ability to study spatiotemporal aspects of receptor activity with great sensitivity and robustness. Validation of the biosensor platform was performed using EGFR but is applicable to the study of other RTKs. For all biosensors tested, EGFR agonist and inhibitor responses were concentration-dependent and exhibited potencies paralleling those documented in the literature^2,19,20,46–48^, highlighting the reliability of the technology. The use of the RTK biosensor platform can further be applied in imaging, allowing the monitoring of specific interactions between protein partners when they are present in close proximity within the same cellular compartment and the translocation of effectors between compartments, as shown in the current study.

The biosensor platform exhibits a wide range of advantages. To begin, the platform provides both qualitative and quantitative data on receptor activity by offering the ability to interrogate live-cell signaling across a vast array of pathways. This represents a significant improvement over classically used in vitro kinase assays, which are limited in their ability to detect compounds that modulate RTK activity allosterically (via mechanisms that are independent of direct kinase inhibition) and compounds whose activity requires accessory proteins and/or cellular co-factors. These advantages, coupled to the ability to screen multiple pathways, significantly reduce the chances of falsely labeling therapeutically viable molecules as inactive.

In addition, the availability of a wide range of effector-specific biosensors linked to different downstream signaling cascades allows for the identification of ‘‘biased ligands” and “biased signaling”. Biased ligands hold potential therapeutic relevance because of their ability to selectively modulate therapeutic signaling cascades while lacking activity on cascades that may produce deleterious side effects. Ligand-biased signaling has been extensively described for GPCRs^49–53^; yet, this field remains underexplored for RTKs, in part due to an emphasis on the discovery of ATP-competitive catalytic domain inhibitors. Reports of biased signaling and ligands have been reported for IGF-1R^54^. Indeed, EGF and Epiregulin displayed differences (especially in potency) in engaging various signaling effectors at the PM. Such differences, however, do not constitute ligand-biased signaling, which is described as ligand‐dependent activation of certain pathways over others. Relative to EGF, Epiregulin is simply less potent in recruiting the SH2 effectors to the PM to an equal degree across the different effectors for which an EC_50_ can be determined. Thus, with regards to PM engagement of SH2 effectors in our experimental system, Epiregulin is a balanced EGFR ligand that displays lower potency in activating EGFR relative to EGF. However, signaling bias may also occur at a spatiotemporal level, with different ligands promoting distinct signaling events at different cellular compartments. Indeed, we demonstrated that EGF, but not Epiregulin, stimulated effector engagement at the early endosomal compartment. Endosomal RTK signaling and subsequent cellular outcomes, including cell transformation and tumorigenesis, are thought to differ from those outcomes resulting from plasma membrane activity (reviewed in^55–57^). Interestingly, recent structural studies of EGFR have revealed that some EGFR natural ligands, Epiregulin and Epigen, stabilize distinct (i.e., less stable) dimeric conformations of the EGFR extracellular domain relative to EGF^2^. Such differences were linked to distinct biological outputs, with EGF promoting proliferation and Epiregulin and Epigen stimulating differentiation of breast cancer cells. Our biosensor platform will help expand knowledge of RTK signaling outputs from endosomal compartments and their relevance to both pathophysiological and therapeutic outcomes.

Unlike other assays offering receptor-proximal functional readouts, the platform described herein does not require the tagging of receptors. The addition of tags to receptors may influence signaling and/or trafficking. Importantly, tagging of RTKs (especially with fluorescent proteins, like GFP) may result in artefactual receptor dimerization. Such dimerization could be driven by the propensity of fluorescent proteins to naturally dimerize^58,59^. Dimerization of RTKs is a hallmark event in receptor activation; as such, fluorescence protein dimerization may result in false and/or increased basal activation of RTK signaling. The use of untagged receptors also makes the ebBRET-based biosensor platform amenable to the study of endogenously expressed receptors, as shown herein with A-431, HeLa, and MDA-MB-231 cells.

Overexpression of the various effector-specific SH2 domains may interfere with the interactions of RTKs with various endogenous scaffolding and/or signaling proteins. As a result, this may disrupt membrane traffic that is elicited upon EGFR activation and may fail to detect all feedback regulation of EGFR signaling. Furthermore, when using heterologous expressed RTKs and the SH2-based effectors, some of the responses could emanate from favorable stoichiometries that may not exist under physiological conditions. Such profiling represents the coupling possibilities and not necessarily the coupling that will be observed in all cell types. Any couplings observed in such settings require further validation to conclude their physiological relevance in cells or tissues of interest.

The biosensors were further applied to the signaling profiling and pharmacological characterization of various activating mutations frequently found in GBM: EGFR-vIII (N-terminal deletion mutant lacking exons 2–7), EGFR-vIV (C-terminal deletion mutant lacking exons 25–27) and the extracellular point mutant EGFR-G598V^34,60^. In accordance with previous reports, we detected ligand-independent (i.e., constitutive) activity of EGFR-vIII, -vIV, and -G598V mutants with conserved ligand responsiveness for mutants EGFR-vIV and -G598V^29,32,61^. Interestingly, our data suggest mutant-specific patterns of constitutive pathway activation, with EGFR-vIII and -vIV mutants displaying greater selectivity in pathway engagement *versus* a more promiscuous profile for the -G598V mutant. The identification of central signaling axes common to a majority of RTK mutants may favor the development of novel therapeutics targeting shared signaling effectors and mechanisms. Such drugs could thus circumvent issues related to tumor heterogeneity and resulting drug resistance.

Various mutations have been shown to confer drug resistance^62^, such as those used in the present study and found in NSCLC. In fact, EGFR mutations such as EGFR-T790M, -C797S, and -T790M/C797S commonly arise during treatment in clinical settings. As exemplified by the Gefitinib and Osimertinib data in Fig. 8, our biosensor platform allowed the differentiation of drugs with different activity profiles toward different mutations leading to drug resistance. To date, overcoming acquired resistance remains an important challenge as tumors constantly develop new resistance mechanisms that counter successive generations of inhibitors. As mutations present a dynamic landscape, elucidating the complexity of acquired and intrinsic mutation mechanisms, and decrypting the signaling profile of RTK mutations, could help identify and develop improved therapeutic tools and strategies.

In summary, this study highlights the applicability of the RTK biosensor platform to perform in-depth, spatiotemporal characterization of RTK biology and pharmacology. The RTK bioSens-All® platform represents a versatile toolset for the development and characterization of novel “biased” RTK ligands and tyrosine kinase inhibitors effective against various mutations involved in drug resistance.

## Methods

### Ligands

The following compounds were used in this study: human recombinant Epidermal Growth Factor (EGF) (Cedarlane; ON, Canada; cat#CL10504), human recombinant Epiregulin (Cedarlane; ON, Canada; cat# CLCYT609), Gefitinib (Iressa®; Tocris Bioscience, United Kingdom; cat# 3000). and Osimertinib (Tagrisso®; MedChemexpress, NJ, USA; cat# HY-15772A).

### Plasmids

Human pcDNA3.1(+)-EGFR-WT was kindly provided by Dr Michel Bouvier’s lab (IRIC, Montreal University, QC, Canada). Human pcDNA3.1(+)-EGFR-WT was used to generate EGFR mutants by mutagenesis: (i) EGFR-T790M: threonine in position 790 mutated in methionine; (ii) EGFR-C797S: cysteine in position 797 mutated in serine; (iii) EGFR-T790M/C797S: threonine in position 790 mutated in methionine and cysteine in position 797 mutated in serine; (iv) EGFR-G598V: glycine in position 598 mutated in valine; (v) EGFR-vIII, truncated between amino-acids 30 and 297; and (vi) EGFR-vIV, truncated between amino-acids 982 and 1054. Human SH2-domains of effector proteins were synthesized, and the generated synthetic DNA fragments were subcloned downstream the RlucII portion into a pCDNA3.1(+) vector after HindIII/XbaI digestion (Supplementary Fig. 1a, b). All plasmids were generated by Topgenetech (Montreal, QC, Canada). Their amino-acid sequences are presented in Supplementary Fig. 1. Both rGFP-CAAX and rGFP-FYVE constructs were previously described^12^.

### Cell culture

Human embryonic kidney 293 HEK293-SL cells were a gift from Dr Stéphane Laporte’s lab (McGill University, Montreal, QC, Canada)^12^ and human epidermoid carcinoma A-431 cells (cat# CRL-1555), human adenocarcinoma HeLa cells (cat# CRM-CCL-2) and human adenocarcinoma MDA-MB-231 cells (cat# CRM-HTB-26) were obtained from the American Type Culture Collection (ATCC, VA, USA). All cell lines were cultured in a complete medium containing Dulbecco’s Modified Eagle Medium (DMEM; without sodium pyruvate, with 4.5 g/L glucose, without L-glutamine; Wisent, QC, Canada; cat# 319-030-CL) supplemented with 1% penicillin-streptomycin (PS; 100 U/mL penicillin and 100 μg/mL streptomycin; Wisent, QC, Canada; cat# 450-201-EL) and 10% fetal bovine serum (FBS; Wisent, QC, Canada; cat# 090150). Cells were divided twice a week and incubated at 37 °C in a humidified atmosphere with 5% CO_2_.

### Transfection

Forty-eight hours before the BRET experiments, HEK293-SL cells were co-transfected with the receptor and one of each RlucII-SH2 / rGFP-localization biosensors. For each transfection condition, the total amount of transfected DNA was kept constant at 1 µg per mL of cell culture to be transfected; whenever necessary, salmon sperm DNA (Invitrogen, CA, USA; cat# 15632011) was used as a DNA carrier to supplement plasmid DNA (i.e., biosensor and receptor). Plasmids quantities per mL of cell culture were assembled in 150 mM NaCl (pH 7.0) as follows: 250 ng of EGFR-WT or EGFR mutants, 10 ng of RlucII-SH2 construct, and 250 ng of rGFP-CAAX (PM) or rGFP-FYVE (EEs) plasmids. PEI (polyethylenimine 25 kDa linear; PolyScience, IL, USA; cat# 23966), previously diluted in 150 mM NaCl, was used as the transfection agent, and the PEI: DNA ratio (µg:µg) was fixed at 3:1. The PEI-containing solution was added to the DNA solution and the DNA/PEI mixture immediately vortexed for 5 s. The DNA/PEI mixture was incubated for at least 20 min at room temperature to allow for the formation of DNA/PEI complexes. During the incubation, HEK293-SL cells were detached, using Trypsin-0,05% EDTA (Wisent, QC, Canada; cat# 325-542-EL), counted and re-suspended in complete DMEM culture medium containing 2% of FBS to a concentration of 3.5 × 10^5^ cells/mL. At the end of the incubation period, the DNA/PEI mixture was added to the cells. Cells were finally distributed in 96-well plates (White Opaque 96-well /Microplates; Greiner, NC, USA; cat# 655083) at a density of 35,000 cells per well. Cells were maintained in culture for the next 48 h before BRET measurements.

A-431 cells were transfected exactly as described for HEK293-SL above, with the exception that no receptor-coding plasmid was transfected since A-431 cells express high levels of endogenous EGFR. HeLa and MDA-MB-231 were transfected using FuGENE® HD Transfection Reagent (Promega, Madison, WI, USA) and OPTI-MEM®I reduced serum medium (Invitrogen; cat# 31985062) as the DNA and FuGENE® HD dilution agent. Cells were distributed in 96-well plates at a density of 45,000 cells per well and maintained in culture for the next 48 h before BRET measurements.

### Bioluminescence resonance energy transfer (BRET) measurements

At 48 h post-transfection, BRET experiments were performed according to the following protocol. Culture medium was aspirated and replaced with 100 µl of pH 7.0 Hank’s Balanced Salt Solution buffer (HBSS; without red phenol; with sodium bicarbonate, with calcium and magnesium, with HEPES; Wisent, QC, Canada; cat# 319-067CL) per well. Plates were incubated for at least 60 min at room temperature to allow equilibration of the transfected cells in the HBSS buffer.

#### Testing of agonist ligands

Increasing concentrations of test compound (EGF or Epiregulin) were added to each well using the HP D300e digital dispenser (Tecan, Switzerland). Compounds were assayed at 22 concentrations for each biosensor. Right after compound injection, 10 µl of 10 µM of luciferase substrate, e-Coelenterazine Prolume Purple (Methoxy e-CTZ; Nanolight Technologies, AZ, USA; cat# 369), were added to each well to a final concentration of 1 µM. Cells were then incubated with the test compounds at room temperature for 10 min (for measurement of responses at the plasma membrane (PM)) or 60 min (for measurement of responses at early endosomes (EE)) under agitation. BRET readings were collected with a 20 s integration time on a SPARK 10 M plate reader (Tecan, Switzerland) with an energy donor filter (400 nm ± 70 nm) and energy acceptor filter (515 ± 20 nm). BRET values were obtained by calculating the ratio of the light emitted by the energy acceptor (rGFP) over the light emitted by the energy donor (RlucII).

#### Testing of inhibitors

To study the inhibitory effects of Gefitinib at both the PM and EE, increasing Gefitinib concentrations were added to cells (as described above). BRET was recorded 30 min later as a measure of any potential agonist (including inverse agonist) activity of the compounds. Immediately following this initial BRET recording, EGF (EC_80_) was directly added to each well (the same well treated with the inhibitor) using the HP D300e digital dispenser. Cells were then incubated at room temperature for an additional 10 min (for measurement of responses at the PM) or 60 min (for measurement of responses at EE), after which time a second BRET measurement was recorded to assess the inhibitory activity of the compounds. All BRET readings were collected, as previously mentioned, on a SPARK 10 M plate reader.

#### Real-time kinetics of agonist ligands

Prior to ligand addition, cells were incubated with 10 µl of 20 µM e-Coelenterazine Prolume Purple (2 µM final concentration) for 5 min at room temperature to stabilize the luciferase signal. Previously determined EC_80_ of EGF or maximal concentration of Epiregulin was then injected using the HP D300e digital dispenser. BRET values were collected each 30 s for 60 min with a 20 s integration time on the SPARK 10 M plate reader. The same filters as described above were used.

#### Real-time kinetics of inhibition

After a 5-minute incubation with a final concentration of 2 µM Prolume Purple, cells were stimulated with an EC_80_ of EGF, and BRET was recorded every 30 s for 30 min (for measurement of responses at the PM) or 60 min (for measurement of responses at EE). After 30 or 60 min of agonist stimulation, 10 µM of Gefitinib were injected, and BRET measurements were recorded every 30 s for another 30 min. In the case of A-431 cells, the same protocol was followed except for the total time of the PM kinetics assay: after 15 min of agonist stimulation, Gefitinib was added for 15 additional minutes.

### BRET Calculations and analysis

BRET values were obtained by calculating the ratio of the light emitted by the energy acceptor (rGFP; 515 ± 20 nm)) over the light emitted by the energy donor (RlucII; 400 nm ± 70 nm). BRET ratios were then standardized using the equation below and represented as universal BRET (uBRET) values:

$$\begin{array}{c}{{{{{\rm{u}}}}}}{{{{{\rm{BRET}}}}}}=\left(\underline{\left({{{{{\rm{BRET}}}}}}\,{{{{{\rm{ratio}}}}}}-{{{{{\rm{A}}}}}}\right)}\right)* 10000\\ ({{{{{\rm{B}}}}}}-{{{{{\rm{A}}}}}})\end{array}$$

Constants A and B, obtained on the SPARK 10 M plate reader, correspond to the following values:

A = pre-established BRET ratio obtained from transfection of a background control (vector coding for RlucII alone).

B = pre-established BRET ratio obtained from transfection of positive control (vector coding for a GFP10-RlucII fusion protein).

The standardized BRET ratio is referred to as uBRET. uBRET data are expressed as the mean of at least three independent experiments ± SEM.

In order to compare basal (constitutive) activities between EGFR-WT and EGFR mutants (Figs. 6c and 7b), a control condition with no receptor and RlucII-SH2 / rGFP-CAAX (for PM) or rGFP-FYVE (for EE) transfected cells was added on each assay plate; using the mean of these ‘biosensor only’ controls, raw uBRET data were normalized and thus allowing to differentiate changes in basal activities due to EGFR mutations. Using the normalized values calculated for non-stimulated conditions, we generated histograms for constitutive activities comparison (vs.EGFR-WT).

### Statistics and reproducibility

All experiments were performed in at least three biological replicates (see figure legends), and data are expressed as the mean of at least three independent experiments ± standard error of the mean (SEM). The statistical significance of the differences was tested using an ordinary one-way ANOVA with Bonferroni post hoc adjustment for multiple comparisons, using GraphPad Prism 8.0. A value of *p* < 0.05 was considered significant. All generated concentration-response curves were fitted and analyzed using the four-parameter logistic non-linear regression model in GraphPad Prism (v8.0, GraphPad Software Inc, CA, USA). Kinetics were fitted using the non-linear one-phase association model in Prism 8.

### BRET imaging

BRET imaging was performed using a BRET microscope composed of an inverted microscope (Eclipse Ti-U, Nikon, NY, USA), an optical filter unit (Lambda 10-2, Sutter Instrument, CA, USA), and an EMCCD camera (HNü512, Nüvü cameras, QC, Canada) as described in Kobayashi et al.^63^.

HEK293-SL cells were seeded 72 h prior measurement on poly-d-lysine-coated 35 mm glass bottom dishes (MatTek, MA, USA; cat# P35GC-1.5-14-C) at a density of 2–6 × 10^5^ cells per dish, and transfected at 48 h before BRET imaging with 250 ng EGFR-WT, 30 ng of RlucII-SH2(Grb2) construct, 250 ng of rGFP-CAAX (PM) or rGFP-FYVE (EEs) plasmids, completed to 1 µg final with ssDNA.

Just before the imaging experiment, cells were washed with pH 7.4 Modified HBSS (137.9 mM NaCl, 5.33 mM KCl, 1 mM CaCl_2_, 1 mM MgCl_2_, 0.44 mM KH_2_PO_4_, 0.33 mM Na_2_HPO_4_, 10 mM HEPES). EGF (10 nM), Gefitinib (1 µM), and the luciferase substrate Coelenterazine-400a (10 µM; Nanolight Technologies, AZ, USA; cat# 340) were diluted in HBSS.

After the addition of the luciferase substrate, photon counting frames were continuously obtained and integrated for 10 s without a filter (total luminescence frames), then for 10 s with a long-pass filter (480LP, acceptor frames). The video represents the time-lapse recording with exposure of 50 s/channel/frame and a video rate of 6FPS (1 s → 300 s). Each frame was recorded with an EM gain of 3000 and 100-ms exposure time. Acceptor and total luminescence images were generated by repeating 25 integration cycles (total exposure time 250 s/channel) and integrating all frames with the same filter settings. Image analysis was performed using MATLAB 2019b (The MathWorks, Inc.). Images were treated with photometric correction^64^ and iterative Poisson image denoising^65^ filters. BRET levels used in BRET imaging correspond to the ratio of acceptor (rGFP) photon counts to donor photon counts calculated for each pixel^63,66^. BRET levels are expressed as a color-coded heat map, with the lowest being black and purple and the highest red and white.

### Flow cytometric evaluation of EGFR expression

For evaluation of cell surface EGFR levels, samples were prepared according to BD Biosciences’ protocol for cell surface staining of stem cells and other adherent cells for flow cytometry. Briefly, cells were transfected as described above, washed once, and incubated in 2% EDTA in PBS solution for 10 min. Cells were then put in suspension by gently pipetting up and down, washed with four volumes of PBS, and once with stain buffer (1xPBS, BSA 2%, NaN3 0.1% pH 7.4), and resuspended to a concentration of 1 × 10^6^ cells/mL. Samples were then fixed by incubating in paraformaldehyde (4% in PBS) for 30 min. The cells were incubated for 1 h on ice with 5 µg/mL antibodies: PE mouse anti-Human EGF Receptor (BD Pharmigen^TM^, ON, Canada; cat # 566778) or PE mouse IgG1 k-Isotype Control (BD Pharmigen^TM^, ON, Canada; cat# 554680). To evaluate total EGFR expression, fixed cells were permeabilized for 20 min in 0.5% Tween-20 solution in PBS. Samples were washed with stain buffer (1xPBS, BSA 2%, NaN3 0.1% pH 7.4) and incubated for 1 h on ice with 0.2 µg antibodies (listed above). Cells were washed twice with stain buffer, and events were recorded on an LSRFortessa™ Cell Analyzer (BD Biosciences). Flow cytometry analysis was performed using the FlowJo v10.10.0 software (BD Biosciences).

### Reporting summary

Further information on research design is available in the Nature Portfolio Reporting Summary linked to this article.

### Supplementary information

Supplementary information

Description of Supplementary Materials

Supplementary Movie 1

Supplementary Data 1 NEW

Reporting Summary

## Data Availability

The amino acid sequences of all RLucII-SH2 effectors and human EGFR-WT and six studied EGFR mutants are shown in Supplementary Fig. 1. Expression levels of EGFR-WT or mutants overexpressed in HEK293 cells are shown in Supplementary Fig. 2. The gating strategy for flow cytometry data is provided as Supplementary Fig. 3. Time-lapse recording of BRET signal for the recruitment of RlucII-SH2(Grb2) at the plasma membrane is shown in Supplementary Movie 1. The source data behind the graphs in the manuscript are shown in Supplementary Data 1.

## References

[1] Lemmon MA, Schlessinger J (2010). Cell signaling by receptor tyrosine kinases. Cell.

[2] Freed DM (2017). EGFR ligands differentially stabilize receptor dimers to specify signaling kinetics. Cell.

[3] Bareja A, Patel S, Hodgkinson CP, Payne A, Dzau VJ (2018). Understanding the mechanism of bias signaling of the insulin-like growth factor 1 receptor: Effects of LL37 and HASF. Cell Signal..

[4] Smith JS, Lefkowitz RJ, Rajagopal S (2018). Biased signalling: from simple switches to allosteric microprocessors. Nat. Rev. Drug Discov..

[5] Crudden C (2018). Blurring boundaries: receptor tyrosine kinases as functional G protein-coupled receptors. Int. Rev. Cell Mol. Biol..

[6] Costa-Neto CM, Parreiras-E-Silva LT, Bouvier M (2016). A pluridimensional view of biased agonism. Mol. Pharm..

[7] Kenakin T (2019). Biased receptor signaling in drug discovery. Pharm. Rev..

[8] Wright SC, Bouvier M (2021). Illuminating the complexity of GPCR pathway selectivity—advances in biosensor development. Curr. Opin. Struct. Biol..

[9] Ménard L, Parker PJ, Kermorgant S (2014). Receptor tyrosine kinase c-Met controls the cytoskeleton from different endosomes via different pathways. Nat. Commun..

[10] Song J, Kwon Y, Kim S, Lee SK (2015). Antitumor activity of phenanthroindolizidine alkaloids is associated with negative regulation of Met endosomal signaling in renal cancer cells. Chem. Biol..

[11] Tan X, Lambert PF, Rapraeger AC, Anderson RA (2016). Stress-induced EGFR trafficking: mechanisms, functions, and therapeutic implications. Trends Cell Biol..

[12] Namkung Y (2016). Monitoring G protein-coupled receptor and β-arrestin trafficking in live cells using enhanced bystander BRET. Nat. Commun..

[13] Molinari P, Casella I, Costa T (2008). Functional complementation of high-efficiency resonance energy transfer: a new tool for the study of protein binding interactions in living cells. Biochem. J..

[14] Pawson T, Gish GD, Nash P (2001). SH2 domains, interaction modules and cellular wiring. Trends Cell Biol..

[15] Liu BA (2006). The human and mouse complement of SH2 domain proteins-establishing the boundaries of phosphotyrosine signaling. Mol. Cell.

[16] Siddiqui S, Cong WN, Daimon CM, Martin B, Maudsley S (2013). BRET biosensor analysis of receptor tyrosine kinase functionality. Front. Endocrinol. (Lausanne).

[17] Henriksen L, Grandal MV, Knudsen SL, van Deurs B, Grovdal LM (2013). Internalization mechanisms of the epidermal growth factor receptor after activation with different ligands. PLoS ONE.

[18] Roepstorff K (2009). Differential effects of EGFR ligands on endocytic sorting of the receptor. Traffic.

[19] Wakeling AE (2002). ZD1839 (Iressa): an orally active inhibitor of epidermal growth factor signaling with potential for cancer therapy. Cancer Res..

[20] Ono M (2004). Sensitivity to gefitinib (Iressa, ZD1839) in non-small cell lung cancer cell lines correlates with dependence on the epidermal growth factor (EGF) receptor/extracellular signal-regulated kinase 1/2 and EGF receptor/Akt pathway for proliferation. Mol. Cancer Ther..

[21] Mellman I, Yarden Y (2013). Endocytosis and cancer. Cold Spring Harb. Perspect. Biol..

[22] Stasyk T, Huber LA (2016). Spatio-temporal parameters of endosomal signaling in cancer: implications for new treatment options. J. Cell Biochem..

[23] Sung M (2018). Caveolae-mediated endocytosis as a novel mechanism of resistance to trastuzumab emtansine (T-DM1). Mol. Cancer Ther..

[24] Carr MT (2019). Comorbid medical conditions as predictors of overall survival in glioblastoma patients. Sci. Rep..

[25] Taylor TE, Furnari FB, Cavenee WK (2012). Targeting EGFR for treatment of glioblastoma: molecular basis to overcome resistance. Curr. Cancer Drug Targets.

[26] Voldborg BR, Damstrup L, Spang-Thomsen M, Poulsen HS (1997). Epidermal growth factor receptor (EGFR) and EGFR mutations, function and possible role in clinical trials. Ann. Oncol..

[27] An Z, Aksoy O, Zheng T, Fan QW, Weiss WA (2018). Epidermal growth factor receptor and EGFRvIII in glioblastoma: signaling pathways and targeted therapies. Oncogene.

[28] Pines G, Kostler WJ, Yarden Y (2010). Oncogenic mutant forms of EGFR: lessons in signal transduction and targets for cancer therapy. FEBS Lett..

[29] Lee JC (2006). Epidermal growth factor receptor activation in glioblastoma through novel missense mutations in the extracellular domain. PLoS Med..

[30] Vivanco I (2012). Differential sensitivity of glioma- versus lung cancer-specific EGFR mutations to EGFR kinase inhibitors. Cancer Discov..

[31] Huang PH, Xu AM, White FM (2009). Oncogenic EGFR signaling networks in glioma. Sci. Signal..

[32] Prigent SA (1996). Enhanced tumorigenic behavior of glioblastoma cells expressing a truncated epidermal growth factor receptor is mediated through the Ras-Shc-Grb2 pathway. J. Biol. Chem..

[33] Gazdar, A. F. Activating and resistance mutations of EGFR in non-small-cell lung cancer: role in clinical response to EGFR tyrosine kinase inhibitors. *Oncogene* **28****Suppl 1**, S24–S31 (2009). 10.1038/onc.2009.198 PMC2849651 19680293

[34] Binder ZA (2018). Epidermal growth factor receptor extracellular domain mutations in glioblastoma present opportunities for clinical imaging and therapeutic development. Cancer Cell.

[35] Gan HK, Kaye AH, Luwor RB (2009). The EGFRvIII variant in glioblastoma multiforme. J. Clin. Neurosci..

[36] AACR Project GENIE: Powering Precision Medicine through an International Consortium. *Cancer Discov.* **7**, 818–831 (2017). 10.1158/2159-8290.CD-17-0151 PMC5611790 28572459

[37] Levkowitz G (1998). c-Cbl/Sli-1 regulates endocytic sorting and ubiquitination of the epidermal growth factor receptor. Genes Dev..

[38] Thien CB, Langdon WY (2001). Cbl: many adaptations to regulate protein tyrosine kinases. Nat. Rev. Mol. Cell Biol..

[39] Huang F, Goh LK, Sorkin A (2007). EGF receptor ubiquitination is not necessary for its internalization. Proc. Natl Acad. Sci. USA.

[40] Jia Y (2016). Overcoming EGFR(T790M) and EGFR(C797S) resistance with mutant-selective allosteric inhibitors. Nature.

[41] Girard N (2018). Optimizing outcomes in EGFR mutation-positive NSCLC: which tyrosine kinase inhibitor and when?. Fut. Oncol..

[42] Suda K, Onozato R, Yatabe Y, Mitsudomi T (2009). EGFR T790M mutation: a double role in lung cancer cell survival?. J. Thorac. Oncol..

[43] Ko B, Paucar D, Halmos B (2017). EGFR T790M: revealing the secrets of a gatekeeper. Lung Cancer (Auckl.).

[44] Wang S, Tsui ST, Liu C, Song Y, Liu D (2016). EGFR C797S mutation mediates resistance to third-generation inhibitors in T790M-positive non-small cell lung cancer. J. Hematol. Oncol..

[45] Thress KS (2015). Acquired EGFR C797S mutation mediates resistance to AZD9291 in non-small cell lung cancer harboring EGFR T790M. Nat. Med..

[46] Wilson KJ (2012). EGFR ligands exhibit functional differences in models of paracrine and autocrine signaling. Growth Factors.

[47] Knudsen SL, Mac AS, Henriksen L, van Deurs B, Grovdal LM (2014). EGFR signaling patterns are regulated by its different ligands. Growth Factors.

[48] Ronan T (2016). Different Epidermal Growth Factor Receptor (EGFR) agonists produce unique signatures for the recruitment of downstream signaling proteins. J. Biol. Chem..

[49] Ramachandran R (2009). Agonist-biased signaling via proteinase activated receptor-2: differential activation of calcium and mitogen-activated protein kinase pathways. Mol. Pharm..

[50] Moller D (2017). Discovery of G protein-biased dopaminergics with a pyrazolo[1,5-a]pyridine substructure. J. Med. Chem..

[51] Wootten D, Christopoulos A, Marti-Solano M, Babu MM, Sexton PM (2018). Mechanisms of signalling and biased agonism in G protein-coupled receptors. Nat. Rev. Mol. Cell Biol..

[52] Wisler JW, Rockman HA, Lefkowitz RJ, Biased G (2018). Protein-coupled receptor signaling: changing the paradigm of drug discovery. Circulation.

[53] Ehrlich AT (2019). Biased signaling of the Mu opioid receptor revealed in native neurons. iScience.

[54] Suleymanova N (2017). Functional antagonism of β-arrestin isoforms balance IGF-1R expression and signalling with distinct cancer-related biological outcomes. Oncogene.

[55] Bergeron JJ, Di Guglielmo GM, Dahan S, Dominguez M, Posner BI (2016). Spatial and temporal regulation of receptor tyrosine kinase activation and intracellular signal transduction. Annu. Rev. Biochem..

[56] Sugiyama MG, Fairn GD, Antonescu CN (2019). Akt-ing up just about everywhere: compartment-specific akt activation and function in receptor tyrosine kinase signaling. Front. Cell Dev. Biol..

[57] Bakker J, Spits M, Neefjes J, Berlin I (2017). The EGFR odyssey—from activation to destruction in space and time. J. Cell Sci..

[58] Snapp EL (2003). Formation of stacked ER cisternae by low affinity protein interactions. J. Cell Biol..

[59] Zacharias DA, Violin JD, Newton AC, Tsien RY (2002). Partitioning of lipid-modified monomeric GFPs into membrane microdomains of live cells. Science.

[60] Furnari FB, Cloughesy TF, Cavenee WK, Mischel PS (2015). Heterogeneity of epidermal growth factor receptor signalling networks in glioblastoma. Nat. Rev. Cancer.

[61] Pines G, Huang PH, Zwang Y, White FM, Yarden Y (2010). EGFRvIV: a previously uncharacterized oncogenic mutant reveals a kinase autoinhibitory mechanism. Oncogene.

[62] Remon J, Steuer CE, Ramalingam SS, Felip E (2018). Osimertinib and other third-generation EGFR TKI in EGFR-mutant NSCLC patients. Ann. Oncol..

[63] Kobayashi H, Picard LP, Schonegge AM, Bouvier M (2019). Bioluminescence resonance energy transfer-based imaging of protein-protein interactions in living cells. Nat. Protoc..

[64] Basden, A. G., Vol. 345. (ed. C. A. Haniff) 985–991 (Mon. Not. R. Astron. Soc., 2003).

[65] Azzari, L., Vol. 23. (ed. A. Foi) <strong _ngcontent-wjb-c27=“”> 1086 - 1090 (IEEE Signal Processing Letters; 2016).

[66] Avet C (2022). Effector membrane translocation biosensors reveal G protein and βarrestin coupling profiles of 100 therapeutically relevant GPCRs. Elife.

