# Supplementary material for: EGFR signaling and pharmacology in oncology revealed with innovative BRET-based biosensors
Source: Commun Biol. 2024 Mar 1;7:250. doi: 10.1038/s42003-024-05965-5 (PMC10907714; doi:10.1038/s42003-024-05965-5)
Supplement: Supplementary file 2 — Description of Supplementary Materials [file 42003_2024_5965_MOESM2_ESM.docx]

**Description of Additional Supplementary Files**

**File name:** Supplementary Data 1

**Description:** The source data behind the graphs in the paper

**File name:** Supplementary Movie 1

**Description:** Time lapse recording of BRET signal for the recruitment of RlucII-SH2(Grb2) at the plasma membrane and inhibition with Gefitinib. Scale bars, 20 µm.
