# Supplementary material for: EGFR signaling and pharmacology in oncology revealed with innovative BRET-based biosensors
Source: Commun Biol. 2024 Mar 1;7:250. doi: 10.1038/s42003-024-05965-5 (PMC10907714; doi:10.1038/s42003-024-05965-5)
Supplement: Supplementary file 5 — Reporting Summary [file 42003_2024_5965_MOESM5_ESM.pdf]

nature portfolio

Corresponding author(s): Laurent Sabbagh

Last updated by author(s): Feb 7, 2024

Reporting Summary

Nature Portfolio wishes to improve the reproducibility of the work that we publish. This form provides structure for consistency and transparency in reporting. For further information on Nature Portfolio policies, see our Editorial Policies and the Editorial Policy Checklist.

Statistics

For all statistical analyses, confirm that the following items are present in the figure legend, table legend, main text, or Methods section.

n/a	Confirmed	
☐	☒	The exact sample size (n) for each experimental group/condition, given as a discrete number and unit of measurement
☐	☒	A statement on whether measurements were taken from distinct samples or whether the same sample was measured repeatedly
☐	☒	The statistical test(s) used AND whether they are one- or two-sided<br>*Only common tests should be described solely by name; describe more complex techniques in the Methods section.*
☒	☐	A description of all covariates tested
☒	☐	A description of any assumptions or corrections, such as tests of normality and adjustment for multiple comparisons
☐	☒	A full description of the statistical parameters including central tendency (e.g. means) or other basic estimates (e.g. regression coefficient) AND variation (e.g. standard deviation) or associated estimates of uncertainty (e.g. confidence intervals)
☐	☒	For null hypothesis testing, the test statistic (e.g. F, t, r) with confidence intervals, effect sizes, degrees of freedom and P value noted<br>*Give P values as exact values whenever suitable.*
☒	☐	For Bayesian analysis, information on the choice of priors and Markov chain Monte Carlo settings
☒	☐	For hierarchical and complex designs, identification of the appropriate level for tests and full reporting of outcomes
☒	☐	Estimates of effect sizes (e.g. Cohen's d, Pearson's r), indicating how they were calculated

Our web collection on statistics for biologists contains articles on many of the points above.

Software and code

Policy information about availability of computer code

Data collection	No software was used
Data analysis	GraphPad Prism (v8.0, GraphPad Software Inc, CA, USA); Image analysis was performed using MATLAB 2019b (The MathWorks, Inc.); Flow cytometry analysis was performed using the FlowJo v10.10.0 software (BD Biosciences).

For manuscripts utilizing custom algorithms or software that are central to the research but not yet described in published literature, software must be made available to editors and reviewers. We strongly encourage code deposition in a community repository (e.g. GitHub). See the Nature Portfolio guidelines for submitting code & software for further information.

Data

Policy information about availability of data

All manuscripts must include a data availability statement. This statement should provide the following information, where applicable:

- Accession codes, unique identifiers, or web links for publicly available datasets
- A description of any restrictions on data availability
- For clinical datasets or third party data, please ensure that the statement adheres to our policy

The amino acid sequences of all RLucII-SH2 effectors and human EGFR-WT and six studied EGFR mutants are shown in Supplementary Figure 1. Expression levels of EGFR-WT or mutants overexpressed in HEK293 cells are shown in Supplementary Figure 2. The gating strategy for flow cytometry data is provided as Supplementary

information. Time lapse recording of BRET signal for the recruitment of RlucII-SH2(Grb2) at the plasma membrane is shown in Supplementary Movie 1. The source data behind the graphs in the manuscript are shown in Supplementary Data 1.

Human research participants

Policy information about studies involving human research participants and Sex and Gender in Research.

Reporting on sex and gender	*Use the terms sex (biological attribute) and gender (shaped by social and cultural circumstances) carefully in order to avoid confusing both terms. Indicate if findings apply to only one sex or gender; describe whether sex and gender were considered in study design whether sex and/or gender was determined based on self-reporting or assigned and methods used. Provide in the source data disaggregated sex and gender data where this information has been collected, and consent has been obtained for sharing of individual-level data; provide overall numbers in this Reporting Summary. Please state if this information has not been collected. Report sex- and gender-based analyses where performed, justify reasons for lack of sex- and gender-based analysis.*
Population characteristics	*Describe the covariate-relevant population characteristics of the human research participants (e.g. age, genotypic information, past and current diagnosis and treatment categories). If you filled out the behavioural & social sciences study design questions and have nothing to add here, write "See above."*
Recruitment	*Describe how participants were recruited. Outline any potential self-selection bias or other biases that may be present and how these are likely to impact results.*
Ethics oversight	*Identify the organization(s) that approved the study protocol.*

Note that full information on the approval of the study protocol must also be provided in the manuscript.

Field-specific reporting

Please select the one below that is the best fit for your research. If you are not sure, read the appropriate sections before making your selection.

☒ Life sciences ☐ Behavioural & social sciences ☐ Ecological, evolutionary & environmental sciences

For a reference copy of the document with all sections, see nature.com/documents/nr-reporting-summary-flat.pdf

Life sciences study design

All studies must disclose on these points even when the disclosure is negative.

Sample size	All experiments were performed in at least three biological replicates (see figure legends) and data are expressed as mean of at least three independent experiments ± standard error of the mean (SEM).
Data exclusions	No data were excluded from the analyses.
Replication	Experiment were repeated at least 3 times (see figure legends). Reproducibility was achieved and SEM are shown.
Randomization	Not relevant to study. Same cell line was used throughout the study with different treatments.
Blinding	Blinding was not relevant to study since no subjects were used in the study.

Reporting for specific materials, systems and methods

We require information from authors about some types of materials, experimental systems and methods used in many studies. Here, indicate whether each material, system or method listed is relevant to your study. If you are not sure if a list item applies to your research, read the appropriate section before selecting a response.

Materials & experimental systems

n/a	Involved in the study	
☐	☒	Antibodies
☐	☒	Eukaryotic cell lines
☒	☐	Palaeontology and archaeology
☒	☐	Animals and other organisms
☒	☐	Clinical data
☒	☐	Dual use research of concern

Methods

n/a	Involved in the study	
☒	☐	ChIP-seq
☐	☒	Flow cytometry
☒	☐	MRI-based neuroimaging

Antibodies

Antibodies used	anti-Human EGF Receptor (BD PharmigenTM, ON, Canada; cat # 566778) or PE mouse IgG1 k-Isotype Control (BD PharmigenTM, ON, Canada; cat# 554680)
Validation	Primary antibody staining was validated using cells expressing or not human EGFR and mutants following transient transfections of HEK293 cells.

Eukaryotic cell lines

Policy information about cell lines and Sex and Gender in Research

Cell line source(s)	Human embryonic kidney 293 HEK293-SL cells were a gift from Dr Stéphane Laporte's lab (McGill University, Montreal, QC, Canada). human epidermoid carcinoma A-431 cells (cat# CRL-1555), human adenocarcinoma HeLa cells (cat# CRM-CCL-2) and human adenocarcinoma MDA-MB-231 cells (cat# CRM-HTB-26) were obtained from the American Type Culture Collection (ATCC, VA, USA)
Authentication	None of the cell lines were authenticated
Mycoplasma contamination	All cell lines tested negative for mycoplasma contamination.
Commonly misidentified lines (See ICLAC register)	*Name any commonly misidentified cell lines used in the study and provide a rationale for their use.*

Flow Cytometry

Plots

Confirm that:

☒ The axis labels state the marker and fluorochrome used (e.g. CD4-FITC).

☒ The axis scales are clearly visible. Include numbers along axes only for bottom left plot of group (a 'group' is an analysis of identical markers).

☒ All plots are contour plots with outliers or pseudocolor plots.

☒ A numerical value for number of cells or percentage (with statistics) is provided.

Methodology

Sample preparation	For evaluation of cell surface EGFR levels, samples were prepared according to BD Biosciences' protocol for cell surface staining of stem cells and other adherent cells for flow cytometry. Briefly, cells were transfected as described above, washed once, and incubated in 2% EDTA in PBS solution for 10 minutes. Cells were then put in suspension by gently pipetting up and down, washed with four volumes of PBS and once with stain buffer (1xPBS, BSA 2%, NaN3 0.1% pH 7.4), and resuspended to a concentration of 1x106 cells/mL. Samples were then fixed by incubating in paraformaldehyde (4% in PBS) for 30 minutes. The cells were incubated for 1 hour on ice with 5µg/mL antibodies: PE mouse anti-Human EGF Receptor (BD PharmigenTM, ON, Canada; cat # 566778) or PE mouse IgG1 k-Isotype Control (BD PharmigenTM, ON, Canada; cat# 554680). To evaluate total EGFR expression, fixed cells were permeabilized for 20 minutes in 0.5% Tween-20 solution in PBS. Samples were washed with stain buffer (1xPBS, BSA 2%, NaN3 0.1% pH 7.4) and incubated for 1 hour on ice with 0.2µg antibodies (listed above). Cells were washed twice with stain buffer prior to acquisition.
Instrument	Events were recorded on a LSRFortessa™ Cell Analyzer (BD Biosciences).
Software	The flow cytometry results were analyzed using FlowJo™ v10.10.0 Software (BD Life Sciences).
Cell population abundance	Not applicable
Gating strategy	Live cells were selected based on FSC-A and SSC-A profiles and defined by gate R1. Singlets were selected based on FSC-W and FSC-H profiles and defined by gate R2. Percentage of cells expressing EGFR is shown in the R3 gate of histograms representing EGFR positive cells from R1/R2 gates. The gate of EGFR positive cells (overexpression) was defined based on staining beyond pcDNA (empty vector) control population. (see supplementary information).

☒ Tick this box to confirm that a figure exemplifying the gating strategy is provided in the Supplementary Information.
